# Immunomodulatory and Mechanistic Considerations of *Hibiscus sabdariffa* (HS) in Dysfunctional Immune Responses: A Systematic Review

**DOI:** 10.3389/fimmu.2021.550670

**Published:** 2021-05-10

**Authors:** Francis U. Umeoguaju, Benson C. Ephraim-Emmanuel, Joy O. Uba, Grace E. Bekibele, Nwondah Chigozie, Orish Ebere Orisakwe

**Affiliations:** ^1^ World Bank Africa Centre of Excellence in Public Health and Toxicological Research (PUTOR), University of Port Harcourt, Port Harcourt, Nigeria; ^2^ Department of Dental Health Sciences, Ogbia, Bayelsa State College of Health Technology, Otakeme, Nigeria; ^3^ Department of Experimental Pharmacology & Toxicology, Faculty of Pharmacy, University of Port Harcourtt, Port Harcourt, Nigeria

**Keywords:** *Hibiscus sabdariffa*, functional food, polyphenols, *Anthocyanin*, immunomodulatory, Anti-inflammatory, cytokines

## Abstract

*Hibiscus sabdariffa* calyx (HS) water decoction extract is a commonly consumed beverage with various pharmacological properties. This systematic review examines the possible effect of HS intake on immune mediators. The Scopus and PUBMED databases were searched for all human and animal studies that investigated the effect of HS administration on immune related biomarkers. For each of the immune biomarkers, the mean, standard deviation and number of subjects were extracted for both the HS treated and untreated group. These values were used in the computation of standardized mean difference (SMD). Statistical analysis and forest plot were done with R statistical software (version 3.6.1). Twenty seven (27) studies met the eligibility criteria. Twenty two (22) of the studies were used for the meta-analysis which included a total of 1211 subjects. The meta-analysis showed that HS administration significantly lowered the levels of TNF-α (n=10; pooled SMD: -1.55; 95% CI: -2.43, -0.67; P < 0.01), IL-6 (n=11; pooled SMD:-1.09; 95% CI: -1.77, -0.40; P < 0.01), IL-1β (n=7; pooled SMD:-0.62; 95% CI: -1.25, 0.00; P = 0.05), Edema formation (n=4; pooled SMD: -2.29; 95% CI: -4.47, -0.11; P = 0.04), Monocyte Chemoattractant Protein -1 (n=4; pooled SMD: -1.17; 95% CI: -1.78, -0.57; P < 0.01) and Angiotensin converting enzyme cascade (n=6; pooled SMD: -0.91; 95% CI: -1.57, -0.25; P < 0.01). The levels of IL-10 (n=4; pooled SMD: -0.38; 95% CI: -1.67, 0.91; P = 0.56), Interleukin 8 (n=2; pooled SMD:-0.12; 95% CI: -0.76, 0.51; P = 0.71), iNOS (n=2; pooled SMD:-0.69; 95% CI: -1.60, 0.23 P = 0.14) and C- Reactive Protein (n=4; pooled SMD: 0.05; 95% CI: -0.26, 0.36; P = 0.75), were not significantly changed by HS administration. Some of the results had high statistical heterogeneity. HS may be promising in the management of disorders involving hyperactive immune system or chronic inflammation.

## Introduction

*Hibiscus sabdariffa* (HS) is a shrub that belongs to the Malvaceae family (1). It is cultivated in many parts of the world such as in West Africa, South Asia (2), the West Indies, Jamaica, China, and the United States (3). It is also cultivated in Egypt, Saudi Arabia, and Sudan (1). It is popularly called Rosella, Mesta, Krajeab, Zobo, and Sorrel in Australia, India, Thailand, Nigeria, and Latin America, respectively (1). Water decoction from dried HS calyx is usually consumed as a beverage drink (4) and used in folk medicine for the management of diseases such as hypertension, liver disorders (5), pyrexia (1, 5), dyslipidemia, diabetes (6), high blood pressure, liver diseases, ulcers, abscesses, and anemia (1). The fleshy calyces of HS are consumed as vegetables (7) and used in making jellies, wine, cakes, syrup, and colorants (8).

The use of HS calyx extracts in traditional medicine has been justified by many studies. Such studies show that extracts from HS possess anti-oxidative (2, 9), radical scavenging (10, 11), angiotensin converting enzyme 1 (ACE1) inhibitory (12–14) activities. HS also possess antihypertensive, anti-bacterial (1), anti-proliferative, anti-glycemic (9, 15) anti-hyperlipidaemic (15), antiobesity (9, 16), anti-inflammatory, anti-carcinogenic (1, 9), antinociceptive, anti-diarrheal, hepatoprotective (1), and antiproliferative (17) activities.

Various phytochemicals such as organic acids (16) phenolic acids (6), flavones and anthocyanin flavonoids (5), essential oils (18), fatty acids (7), and polysaccharides (4) have been detected in extracts from HS. Major organic acids identified in HS include hydroxycitric acids, hibiscus acid, and its derivatives (16, 17, 19, 20). The predominant phenolic acids in extracts from HS include protocatechuic acid (PCA) (21), 3-O-caffeoylquinic acid (7), chlorogenic acid (6), neochlorogenic acid, methyl digallate, methyl chlorogenate, coumaroilquinic acid, dihyroferulic acid 4-o-glucoronide, 5-O-caffeoylshikimic acid, and ethyl chlorogenate, etc. (19). The aglycones, O, and 3-substituted derivatives of quercetin, myricetin, kaempferol, Gossypetin, and methyl epigallocatechin have also been identified from extracts of HS (7, 19, 20).

Anthocyanins are the major flavonoids present in HS; they are responsible for the color pigment. Anthocyanins are water soluble glycosides or acylglycosides of anthocyanidin which are derivates of the 2-phenylbenzopyrylium salt (5). Delphinidin 3-sambubioside forms about 85% of the anthocyanin contents of HS including cyanidin 3-sambubioside (major pigment), and delphinidin-3-glucoside and cyanidin 3-glucoside as minor pigments (5, 6, 22). Other derivatives of anthocyanins have also been reported (17). The carbohydrates identified from extracts of HS include glucose, galactose, xylose, arabinose, rhamnose, and pectin-like polysaccharides (4, 23). Fatty acids such as hexadecanoic, 14-Methyl-pentadecanoic acid, and methyl esters have also been identified in HS (18). Palmitic acid and alpha tocopherol are the most abundant lipophilic compounds in HS (7).

HS has recently gained attention because it has beneficial effects in the management of hypertension and other metabolic syndromes such as obesity and diabetes. Hypertensive subjects have elevated serum ACE1 activity (24, 25). Accumulating evidence demonstrates the inhibitory activity of HS extract on ACE1 activity (13, 25, 26). The catalytic conversion of angiotensin I (Ang I) to angiotensin II (Ang II) by ACE1 leads to the buildup of Ang II. Elevated levels of inflammatory mediators and the depletion of cellular antioxidant capacity have been observed in different models of hypertension (25, 27–29). Ang II-induced stimulation of the angiotensin II receptor type 1 (AT1 receptor) could lead to the activation of Nuclear Factor Kappa B (NF-κB), bringing about the expression of pro-inflammatory genes (29, 30). ACE1 inhibitors can lower Ang II levels thereby suppressing the Ang II-induced stimulation of AT1 receptors. The antioxidant (2, 21) and ACE1 inhibitory (13, 24) activities of constituents of HS extract, makes it potentially able to suppress inflammatory reactions. HS has been reported to suppress the levels of NF-κB in Lipopolysaccharide (LPS)-stimulated recombinant Human hepatoma cell line (HepG2) cells (31), metabolic syndrome, and Thioacetamide (TAA)-intoxicated rats (17). There is currently no systematic review or meta-analysis on the effect of HS extract supplementation on the expression of inflammatory cytokines in different disease and nondisease models. This review thus aimed to review the available evidence on the immunomodulatory potential of HS extracts and how such activity might be exploited in the management of pathological conditions linked to hyperactive immune responses.

## Methods

This review was done according to the recommendations of the Preferred Reporting Items for Systematic Reviews and Meta-Analyses (PRISMA) (32).

### Data Sources and Search Strategy

Online databases (Pubmed and Scopus) were systematically searched by two researchers reviewing independently (UF and NC) to find articles that documented the impact of HS on immune biomarkers. Date restriction was not applied in the search. The search was conducted between March 20, 2020, and June 5, 2020. The databases were searched with the keywords (“*Hibiscus sabdariffa*” OR roselle). The title and abstract of all the resultant search results were then screened for eligibility.

### Eligibility Criteria

The following were inputted in the inclusion criteria; (1) original articles published in the English language. (2) Human and animal studies that used HS as an intervention agent in a healthy or disease model. (3) Studies that reported any immune or inflammatory related biomarkers such as Interleukins, Chemokines, Toll-like receptors (TLRs), Prostaglandins, Tumor Necrosis Factor-α (TNF-α), NF-κB, Monocyte chemoattractant protein-1 (MCP-1/CCL2), Inducible Nitric Oxide synthase (iNOS), components of the cyclooxygenase and lipoxygenases pathways, components of the ACE cascade (Ang I, Ang II, AT1 receptor, Angiotensin II receptor type 2 (AT2 receptor), ACE), inflammatory endpoints such as edema formation, and inflammatory diseases, etc. (4) Studies done using any extract from the calyces of HS. (5) Only studies that included both a control and an HS-intervention group were considered. Data obtained from *in vitro* studies, grey literature, meta-analysis, and reviews were excluded from the review. Studies done on extracts obtained from parts of HS (other than the calyx) or other species of Hibiscus were excluded.

### Data Selection

Duplicate records were deleted, after which two authors (UF, NC) reviewed the collated articles to ascertain their suitability for inclusion. Where the two authors had different opinions on the inclusion of an article, the other researchers (EEBC, UJ, and BG) voted and a consensus was reached. Titles and abstracts of articles were first screened, and publications that did not meet the written criteria were removed. Five independent researchers (UF, BG, EEBC, UJ, NC) assessed the full texts of the remaining articles for eligibility. For each of the eligible studies, relevant data were extracted independently by each of the reviewers (UF, BG, EEBC, UJ, NC), into a predesigned data extraction table which consisted of the first author’s last name, year of study, experimental model, HS formulation, dosage/route of administration, analytical method, the inflammatory biomarker investigated, tissue investigated, study sample size and summary of major findings. The data extraction table generated by each reviewer was subsequently compared and collated.

### Statistical Analysis

Missing or incomplete data was estimated with Cochrane’s review manager software (RevMan 5.3). Statistics and meta-analysis were done with R statistical software (version 3.6.1). A random effect model was assumed and meta-analysis was computed using the DerSimonian-Laird tau estimator. The forest plot was plotted with the “meta-forest” statistical package using R (33–35). The standardized mean difference (SMD) was used as an index of the effect size. SMD was used for the forest plot since varying units were showing a particular effect across studies (36). The random effect model was assumed to accommodate the different experimental models that generated the investigated effects (36).

The forest plot is essential for analyzing the pooled estimate of the mean differences between the control and the HS intervention group across different studies. The overall standardized mean difference and the pooled 95% confidence interval were presented for each biomarker analyzed. A forest plot was carried out on a particular immune-related biomarker only if more than one study reported complete data for that particular biomarker. The effect of HS on the levels of inflammatory biomarkers across healthy and diseased subgroups of the experimental models, as well as across human and rodent models were examined using subgroup analysis. Raw data for mean and standard deviation (SD) were extracted from chart images with the aid of a digital pixel ruler downloaded from www.arulerforwindows.com.

For studies that reported standard error of mean (SEM) values, the SD was calculated using SD = (SEM∗√n).

In estimating the pooled effect of HS administration on ACE signaling, data from different members of the pathways were combined in one analysis. For example the Ang II, ACE1, and Ang I receptors were all analyzed under ACE signaling. In studies where more than one dose of the HS extract was given, the dose that elicited the most effect was reported and used for the analyses. Some of the studies recorded some missing data. Such missing data (when possible), was estimated using RevMan 5.3. In some cases, the published data were not reported in forms that can be subjected to analysis with other data. Such data were transformed appropriately before analysis.

## Results

### Data Selection

**Figure 1:**
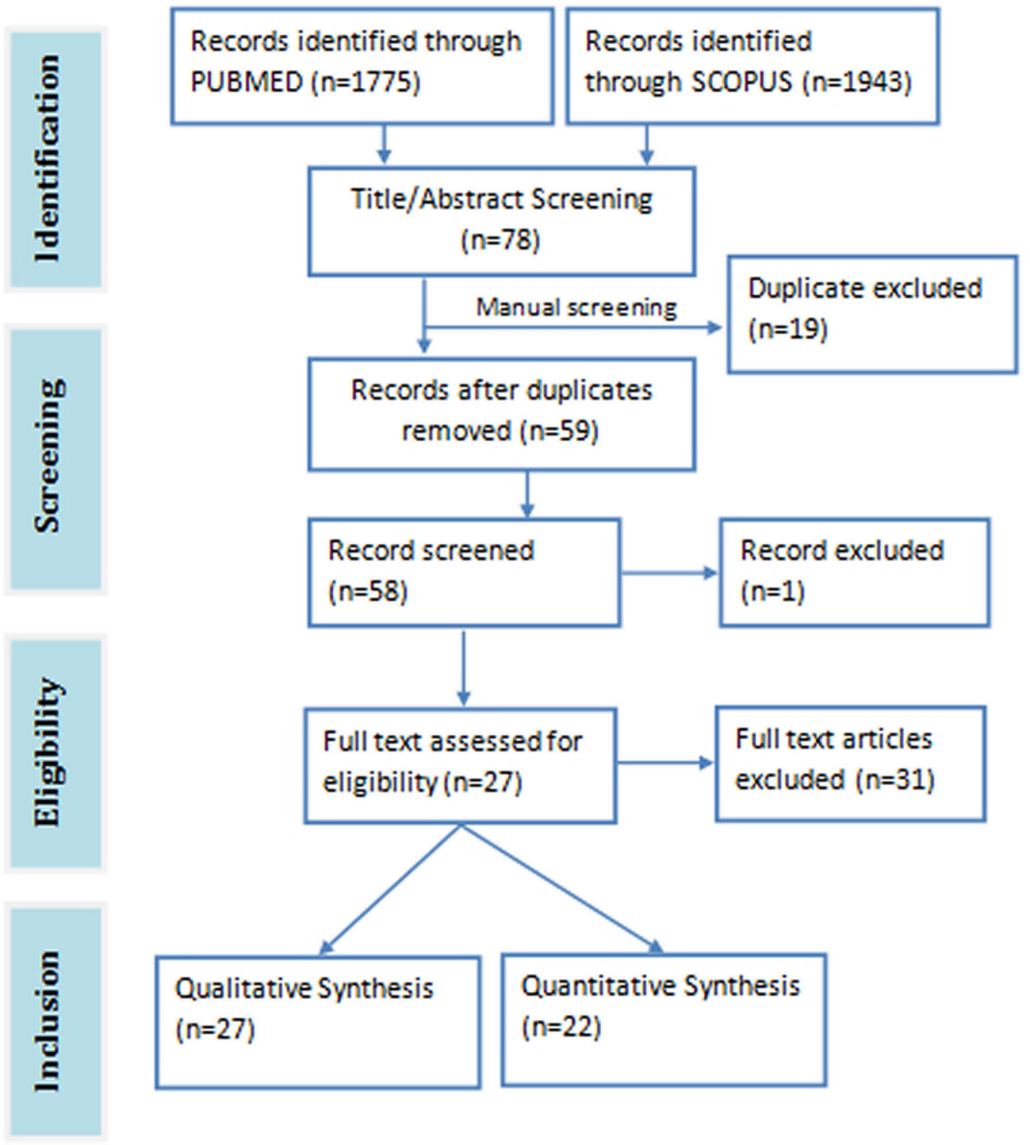
Prima flowchart of included studies.

The total search hits from both databases were 3718 papers (1943 from Scopus and 1775 from PubMed). A total of 78 relevant articles were downloaded after screening the title and abstract of the search results against the eligibility criteria. Duplicates articles (n=19) were removed and the remaining unique articles (n=59) were subjected to full-text screening. One article was excluded on account of being inaccessible. Twenty seven (27) articles were eventually selected for the review study, 22 of which were used for the meta-analysis (**Figure 1**). The meta-analysis consists of three pre-post clinical trials, three randomized double blind clinical trials, one randomized single blind clinical trial, 15 animal studies, and 1211 subjects in all. The details of the included and excluded study are shown in **Table 1**.

**Table 1:** Summary of included and excluded studies.

Included for narrative synthesis	Included for meta-analysis	Excluded from the meta-analysis
(37), (38), (9), (39), (40), (20), (41),(24), (42), (43), (11), (31), (25), (17), (44), (45), (46), (47), (26), (48), (16), (49), (50), (15), (51), (1), (52)	(1), (52), (50), (49), (16), (9), (11), (47), (26), (45), (48), (24), (37), (46), (42), (44), (42), (43), (41), (20), (31) and (17)	Wang et al. (37), reported only qualitative data.
		Kao et al. (38), reported only qualitative data on immune related biomarker.
		Nurkhasanah (39) had missing data (sample size was not stated in the article).
		Abdel-Rahman et al. (25), investigated HS in combination with extract of Olea europaea.
		Lubis et al. (40), did not report their findings as means of observations.

### Effect of HS Extracts on Inflammatory and Immune Mediators

Administration of HS calyces extract significantly decreased the levels of different pro-inflammatory biomarkers such as TNF-α (9, 11, 16, 31, 40–43), Interleukin 6 (IL-6) (11, 16, 17, 40, 42–44), iNOS (31, 45), Toll-like receptor 4 (TLR4) (16), Interleukin 1β (IL-1β) (9, 16, 42), MCP-1 (11, 16, 42), Ang I (46), Ang II (26), ACE1 (24–26, 47), NF-κB (17), and Cyclooxygenase-2 (COX-2) (38, 48). It also significantly decreased the levels of inflammatory manifestations of ear edema (1, 49) and paw edema (11). HS treatments do not seem to have any significant effect on the levels of C-reactive protein (CRP) (42, 50, 51). There are conflicting reports on the effect of HS extracts on the levels of interleukin 10 (IL-10). While Fakeye et al. (41) and Villalpando-Arteaga et al. (9) did not detect any significant difference, Lubis et al. (40), and Nurkhasanah (39) reported a significant increase and Ali et al. (44), reported a significant decrease in IL-10 levels. Some studies did not detect any significant difference in the levels of ACE, IL-6 and IL-1β following treatments with HS calyx extract (9, 14, 42, 52).

### Meta-Analysis of Effects Across Studies

**Figures 2**–**11** show the meta-analysis of the effect of HS extract administration on the levels of Ang II cascade (ACE1, Ang II, Ang I, AT1 receptors), CRP, Edema formation, iNOS, IL-1β, IL-6, Interleukin 8 (IL-8), IL-10, MCP-1, and TNF-α across different studies.

**Figure 2:**
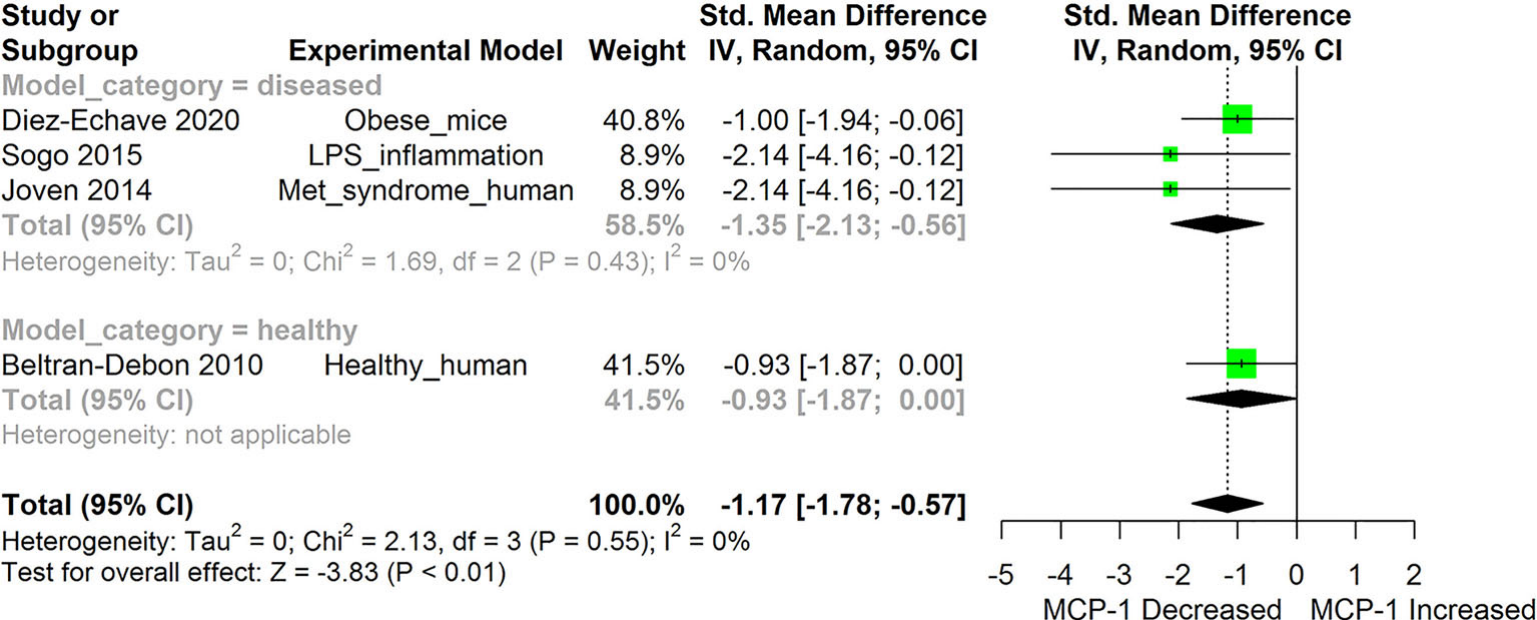
Meta-analysis and forest plot of the effect of HS administration on Monocyte Chemoattractant Protein -1b (MCP-1/CCL2). The meta-analysis was sub-grouped by health status (i.e diseased or healthy state). The standardized mean difference (SMD), which is an estimate of the effect size, is presented as a square on the forest plot, for each study and as a diamond for the pooled estimate of each subgroup or overall studies. The overall pooled SMD for all included studies is shown in the bottom section of the forest plot. SMD data points that are lower than zero signify that treatments with HS lowered the level of the biomarker while those greater than zero indicate that HS treatment causes an increase in the levels of the biomarker. The statistical significance of the pooled estimate is indicated by the Z test of significance. LPS, Lipopolysaccharide; Met, Metabolic.

**Figure 3:**
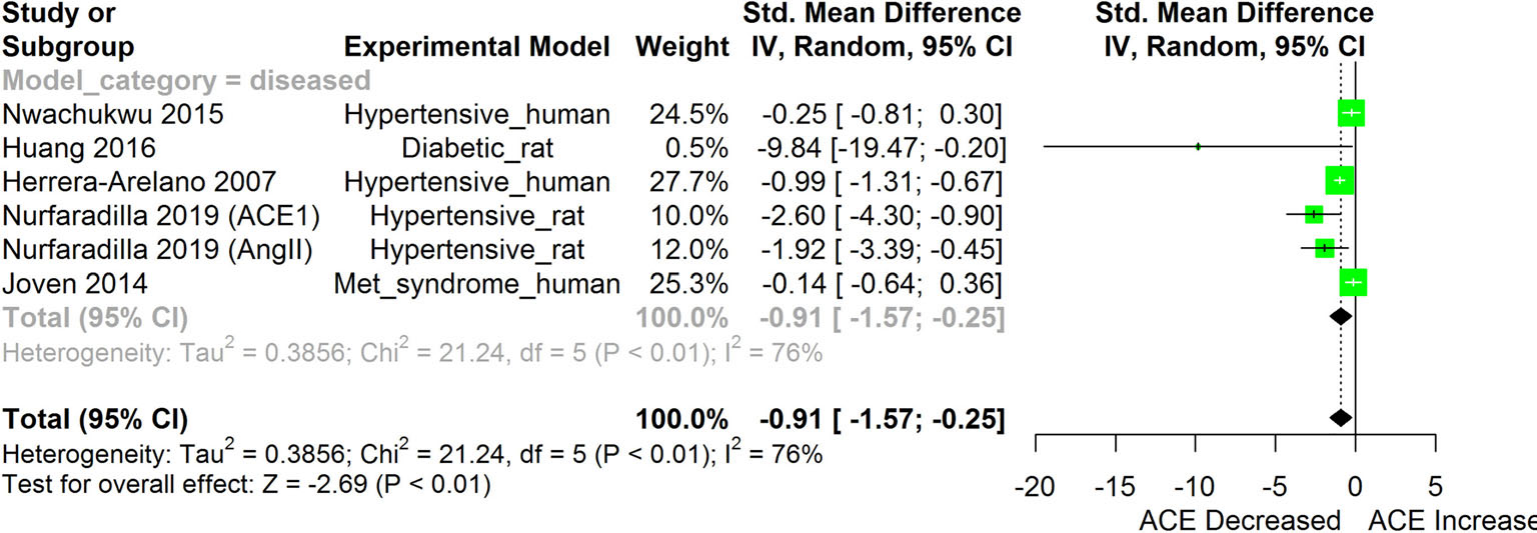
Meta-analysis and forest plot of the effect of HS administration on Angiotensin converting enzyme cascade. The meta-analysis was sub-grouped by health status (i.e diseased or healthy state). The standardized mean difference (SMD), which is an estimate of the effect size, is presented as a square on the forest plot, for each study and as a diamond for the pooled estimate of each subgroup or overall studies. The overall pooled SMD for all included studies is shown in the bottom section of the forest plot. SMD data points that are lower than zero signify that treatments with HS lower the level of the biomarker while those greater than zero signify that HS treatment causes an increase in the levels of the biomarker. The statistical significance of the pooled estimate is indicated by the Z test of significance. Met, Metabolic.

**Figure 4:**
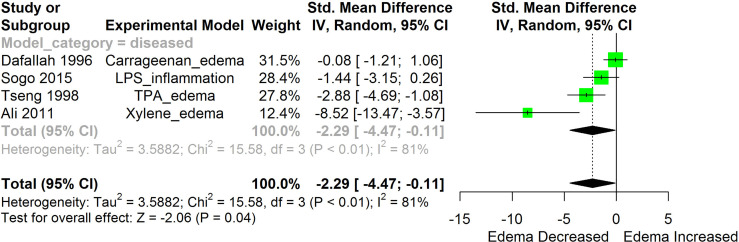
Meta-analysis and forest plot of the effect of HS administration on Edema formation. The meta-analysis was sub-grouped by health status (i.e diseased or healthy state). The standardized mean difference (SMD), which is an estimate of the effect size, is presented as a square on the forest plot, for each study and as a diamond for the pooled estimate of each subgroup or overall studies. The overall pooled SMD for all included studies is shown in the bottom section of the forest plot. SMD data points that are lower than zero signify that treatments with HS lowered the level of the biomarker while those greater than zero signify that HS treatment causes an increase in the levels of the biomarker. The statistical significance of the pooled estimate is indicated by the Z test of significance. LPS, Lipopolysaccharide; TPA, 12-O-tetradecanoylphorbol-13-acetate.

**Figure 5:**
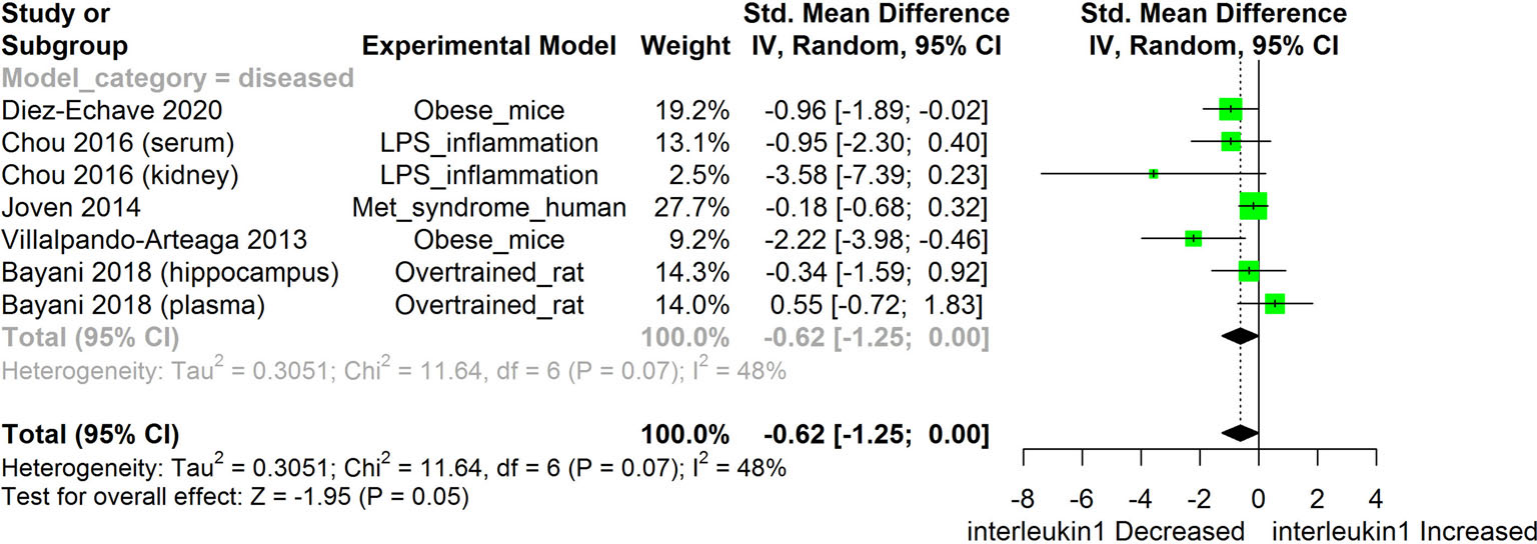
Meta-analysis and forest plot of the effect of HS administration on IL-1β. The meta-analysis was sub-grouped by health status (i.e diseased or healthy state). The standardized mean difference (SMD), which is an estimate of the effect size, is presented as a square on the forest plot, for each study and as a diamond for the pooled estimate of each subgroup or overall studies. The overall pooled SMD for all included studies is shown in the bottom section of the forest plot. SMD data points that are lower than zero signify treatments with HS lowered the level of the biomarker while those greater than zero signifies that HS treatment causes an increase in the levels of the biomarker. The statistical significance of the pooled estimate is indicated by the Z test of significance. LPS, Lipopolysaccharide; Met, Metabolic.

**Figure 6:**
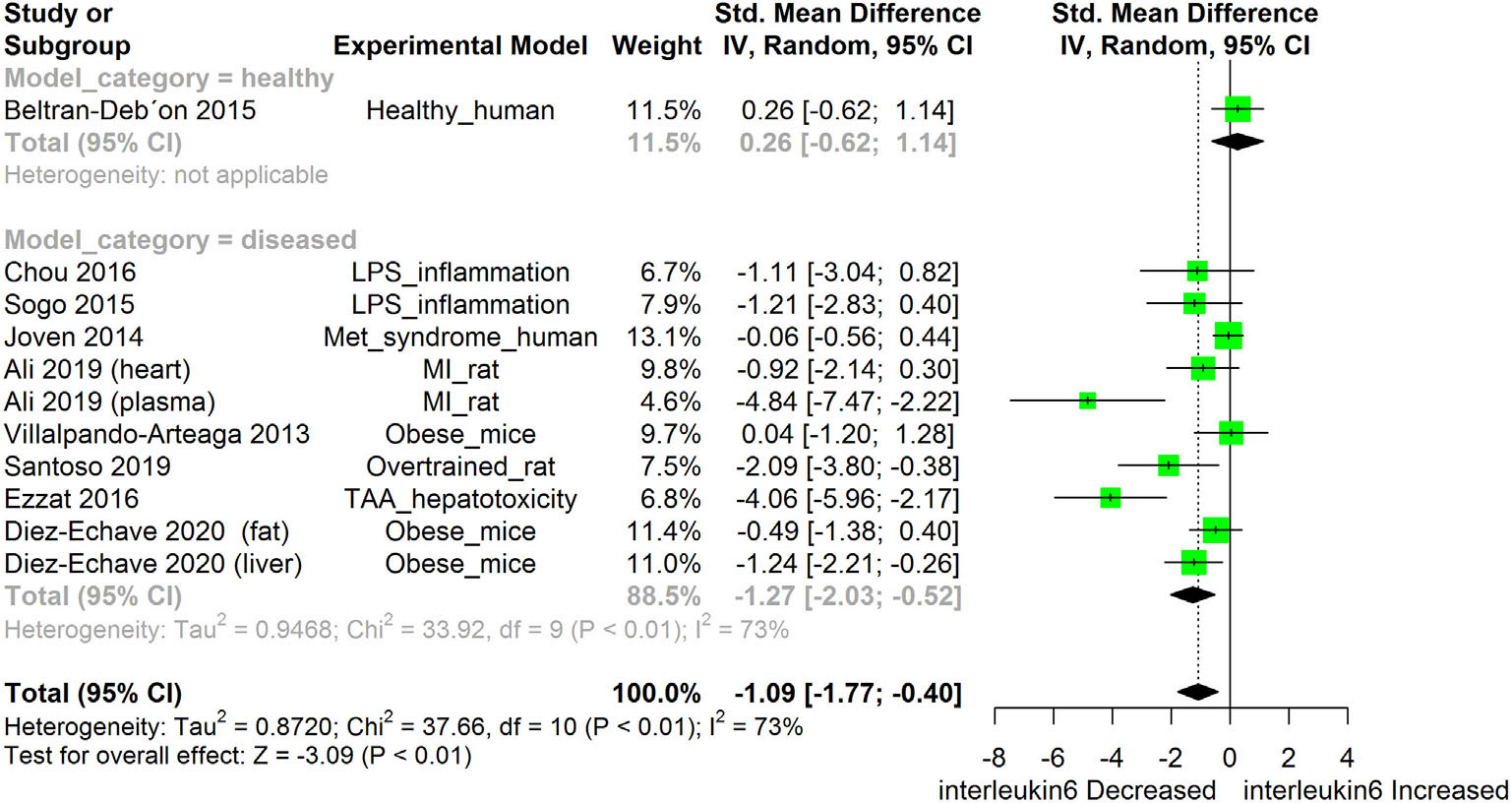
Meta-analysis and forest plot of the effect of HS administration on IL-6. The meta-analysis was sub-grouped by health status (i.e diseased or healthy state). The standardized mean difference (SMD) which is an estimate of the effect size, is presented as a square on the forest plot, for each study and as a diamond for the pooled estimate of each subgroup or overall studies. The overall pooled SMD for all included studies is shown in the bottom section of the forest plot. SMD data points that are lower than zero signify that treatments with HS lowered the level of the biomarker while those greater than zero signify that HS treatment caused an increase in the levels of the biomarker. The statistical significance of the pooled estimate is indicated by the Z test of significance. LPS, Lipopolysaccharide; Met, Metabolic; TAA, Thioacetamide; MI, Myocardiac infarction.

**Figure 7:**
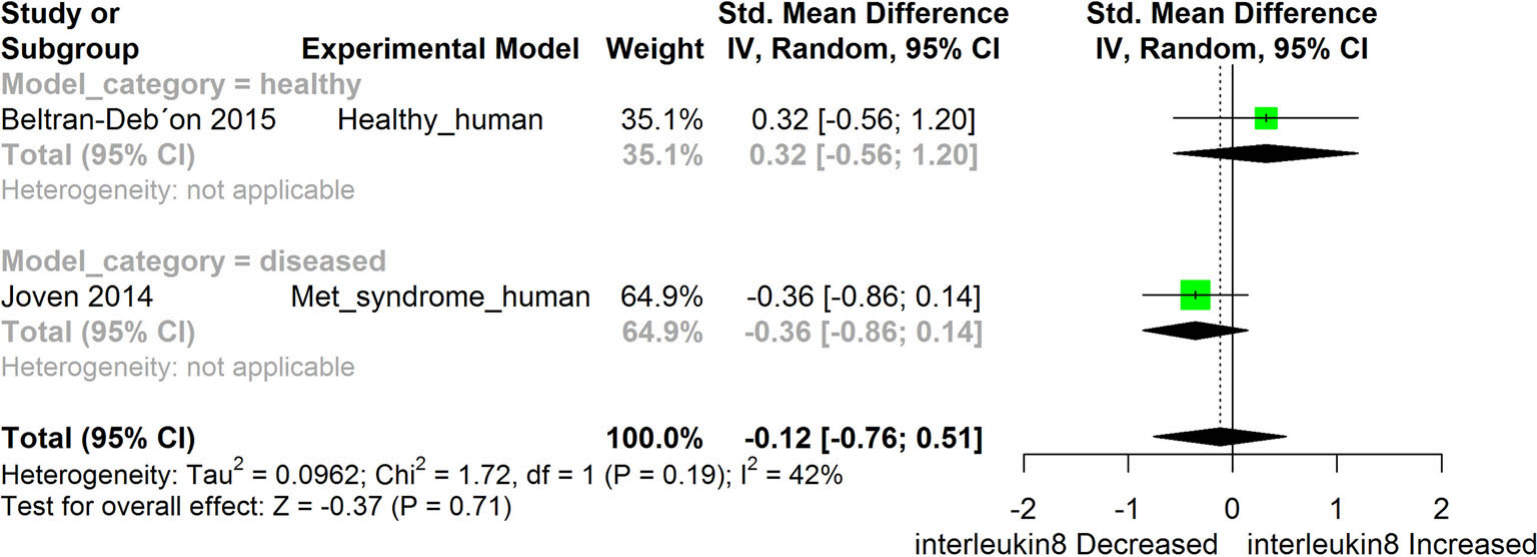
Meta-analysis and forest plot of the effect of HS administration on IL- 8. The meta-analysis was sub-grouped by health status (i.e diseased or healthy state). The standardized mean difference (SMD), which is an estimate of the effect size, is presented as a square on the forest plot, for each study and as a diamond for the pooled estimate of each subgroup or overall studies. The overall pooled SMD for all included studies is shown in the bottom section of the forest plot. SMD data points that are lower than zero signify that treatments with HS lowered the level of the biomarker while those greater than zero signify that HS treatment causes an increase in the levels of the biomarker. The statistical significance of the pooled estimate is indicated by the Z test of significance. Met, Metabolic.

**Figure 8:**
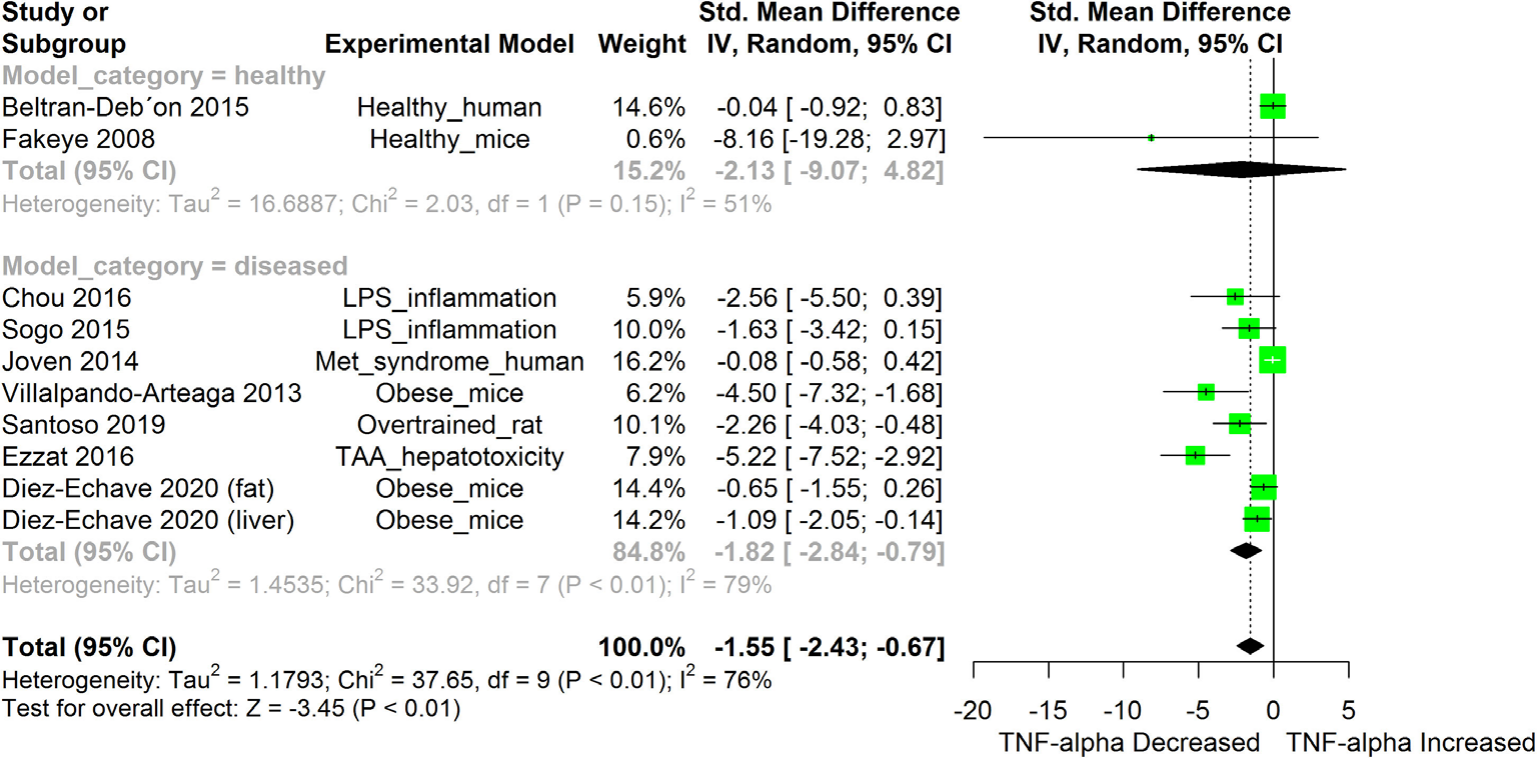
Meta-analysis and forest plot of the effect of HS administration on TNF-α. The meta-analysis was sub-grouped by health status (i.e diseased or healthy state). The standardized mean difference (SMD), which is an estimate of the effect size, is presented as a square on the forest plot, for each study and as a diamond for the pooled estimate of each subgroup or overall studies. The overall pooled SMD for all included studies is shown in the bottom section of the forest plot. SMD data points that are lower than zero signify that treatments with HS lowered the level of the biomarker while those greater than zero signify that HS treatment caused an increase in the levels of the biomarker. The statistical significance of the pooled estimate is indicated by the Z test of significance. LPS, Lipopolysaccharide; Met, Metabolic; TAA, Thioacetamide.

**Figure 9:**
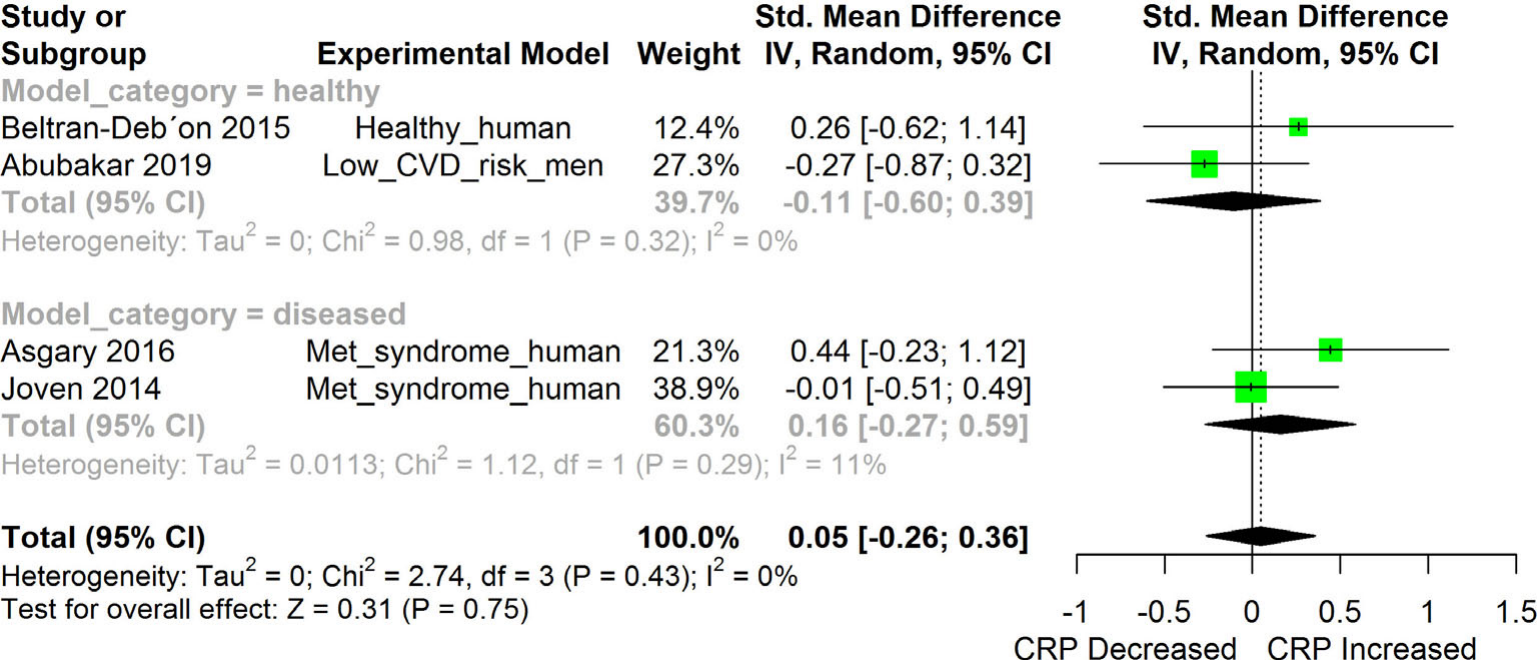
Meta-analysis and forest plot of the effect of HS administration on CRP. The meta-analysis was sub-grouped by health status (i.e diseased or healthy state). The standardized mean difference (SMD), which is an estimate of the effect size, is presented as a square on the forest plot, for each study and as a diamond for the pooled estimate of each subgroup or overall studies. The overall pooled SMD for all included studies is shown in the bottom section of the forest plot. SMD data points that are lower than zero signify that treatments with HS lowered the level of the biomarker while those greater than zero signify that HS treatment causes an increase in the levels of the biomarker. The statistical significance of the pooled estimate is indicated by the Z test of significance. Met, Metabolic.

**Figure 10:**
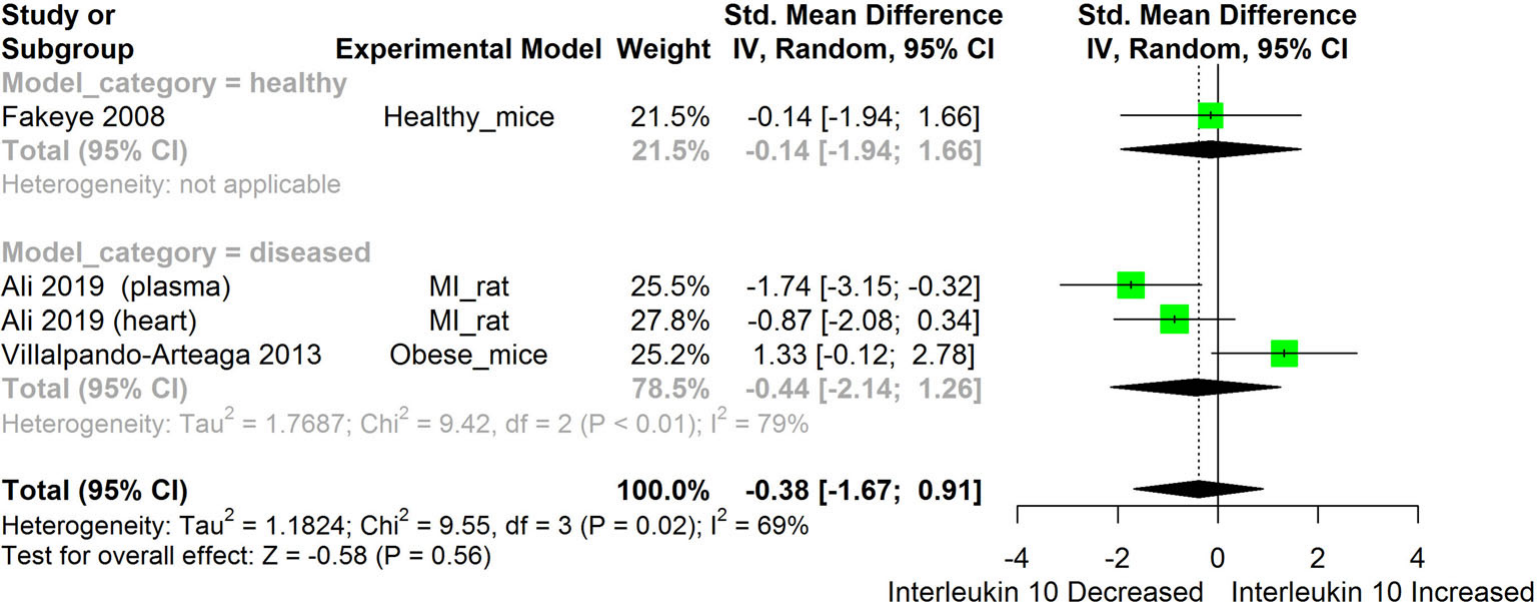
Meta-analysis and forest plot of the effect of HS administration on IL-10. The meta-analysis was sub-grouped by health status (i.e diseased or healthy state). The standardized mean difference (SMD) which is an estimate of the effect size, is presented as a square on the forest plot, for each study and as a diamond for the pooled estimate of each subgroup or overall studies. The overall pooled SMD for all included studies is shown in the bottom section of the forest plot. SMD data points that are lower than zero signify that treatments with HS lowered the level of the biomarker while those greater than zero signify that HS treatment caused an increase in the levels of the biomarker. The statistical significance of the pooled estimate is indicated by the Z test of significance. MI, Myocardiac infarction.

**Figure 11:**
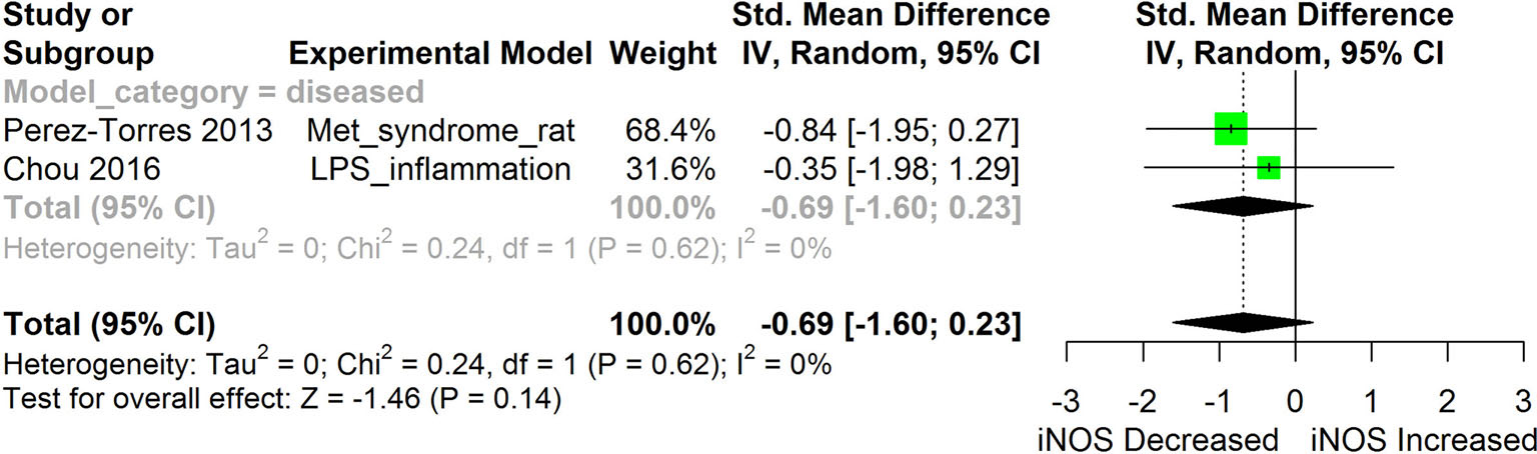
Meta-analysis and forest plot of the effect of HS administration on iNOS. The meta-analysis was sub-grouped by health status (i.e diseased or healthy state). The standardized mean difference (SMD), which is an estimate of the effect size, is presented as a square on the forest plot, for each study and as a diamond for the pooled estimate of each subgroup or overall studies. The overall pooled SMD for all included studies is shown in the bottom section of the forest plot. SMD data points that are lower than zero signify that treatments with HS lowered the level of the biomarker while those greater than zero signify that HS treatment causes an increase in the levels of the biomarker. The statistical significance of the pooled estimate is indicated by the Z test of significance. LPS, Lipopolysaccharide; Met, Metabolic.

HS administration lowered the MCP-1 levels when compared to untreated groups in the studies examined. The pooled SMD showed a significant decrease in MCP-1 (**Figure 2**) following treatments with HS extract (n=4; pooled SMD:-1.17; 95% CI: -1.78, -0.57; P < 0.01). Components of the ACE cascade (**Figure 3**) were lowered significantly following HS administration (n=6; pooled SMD:-0.91; 95% CI: -1.57, -0.25; P < 0.01). Edema formation (**Figure 4**) was significantly reduced following HS treatment (n=4; pooled SMD:-2.29; 95% CI: -4.47, -0.11; P = 0.04). IL-1β (**Figure 5**) was significantly lowered by HS treatment (n=7; pooled SMD:-0.62; 95% CI: -1.25, 0.00; P = 0.05). IL-6 (**Figure 6**) was also lowered significantly (n=11; pooled SMD:-1.09; 95% CI: -1.77, -0.40; P < 0.01). IL-8 was not significantly altered by HS administration (**Figure 7**; n=2; pooled SMD:-0.12; 95% CI: -0.76, 0.51; P = 0.71). TNF-α was significantly reduced following treatments with HS (**Figure 8**: n=10; pooled SMD:-1.55; 95% CI: -2.43, -0.67; P < 0.01). The levels of CRP (**Figure 9**: n=4; pooled SMD: 0.05; 95% CI: -0.26, 0.36; P = 0.75), IL-10 (**Figure 10**: n=4; pooled SMD:-0.38; 95% CI: -1.67, 0.91; P = 0.56), and iNOS (**Figure 11**: n=2; pooled SMD:-0.69; 95% CI: -1.60, 0.23 P = 0.14) were not significantly affected by HS treatment. A high heterogeneity was observed for TNF, IL-6, ACE, and edema formation. The diverse experimental models used in most of the included studies could partly explain the observed heterogeneity. The observed heterogeneity could affect the extrapolation of this meta-analysis to other similar populations (36).

**Figure 12:**
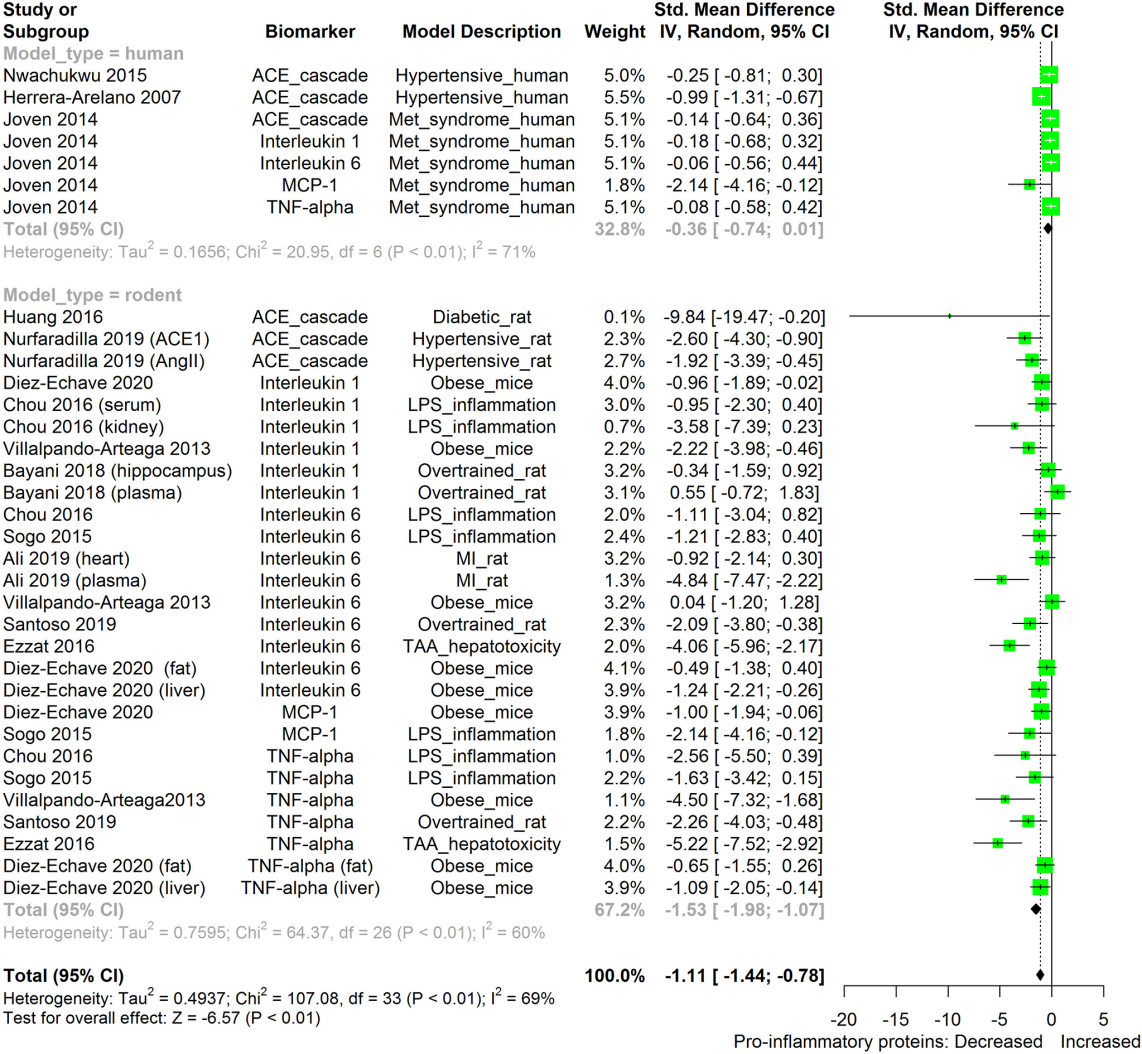
Meta-analysis and forest plot of the effect of HS administration on selected pro-inflammatory biomarkers (ACE, TNF-α, IL-6, IL-1β, MCP-1) from diseased human and rodent models. The meta-analysis was sub-grouped by model organism (i.e diseased human or diseased rodents models). The standardized mean difference (SMD), which is an estimate of the effect size, is presented as a square on the forest plot, for each study and as a diamond for the pooled estimate of each subgroup or overall studies. The overall pooled SMD for all included studies is shown in the bottom section of the forest plot. SMD data points that are lower than zero signify that treatments with HS lowered the level of the biomarker while those greater than zero signify that HS treatment causes an increase in the levels of biomarker. The statistical significance of the pooled estimate is indicated by the Z test of significance. MI, Myocardiac infarction; LPS, Lipopolysaccharide; TAA, Thioacetamide; Met, Metabolic; TNF-alpha, Tumour necrosis factor-alpha; MCP-1, Monocyte chemoattractant protein-1; ACE, Angiotensin-converting enzyme.

The meta-analysis (**Figures 2**–**11**) showed that the levels of TNF-β, IL-6, IL-1β, MCP-1, and ACE were significantly lowered by HS. An attempt was made to compare the efficacy of HS extract in lowering the levels of TNF-a, IL-6, IL-1β, MCP-1, and ACE, in human and animal disease models. This was done by running a subgroup meta-analysis using the biomarkers TNF- α, IL-6, IL-1β, MCP-1, and ACE on diseased models of humans and rodents. The result is shown in **Figure 12**. The result shows that HS extract is more active in suppressing the inflammatory biomarkers in rodents (n=27; pooled SMD:-1.53; 95% CI: -1.98, -1.07; p < 0.01) than in human subjects (n=7; pooled SMD:-0.36; 95% CI: -0.74, 0.01; p=0.06). This result may be due to the comparatively low number of human studies. It may also be due to the limited experimental models reported in available human studies.

**Figure 13:**
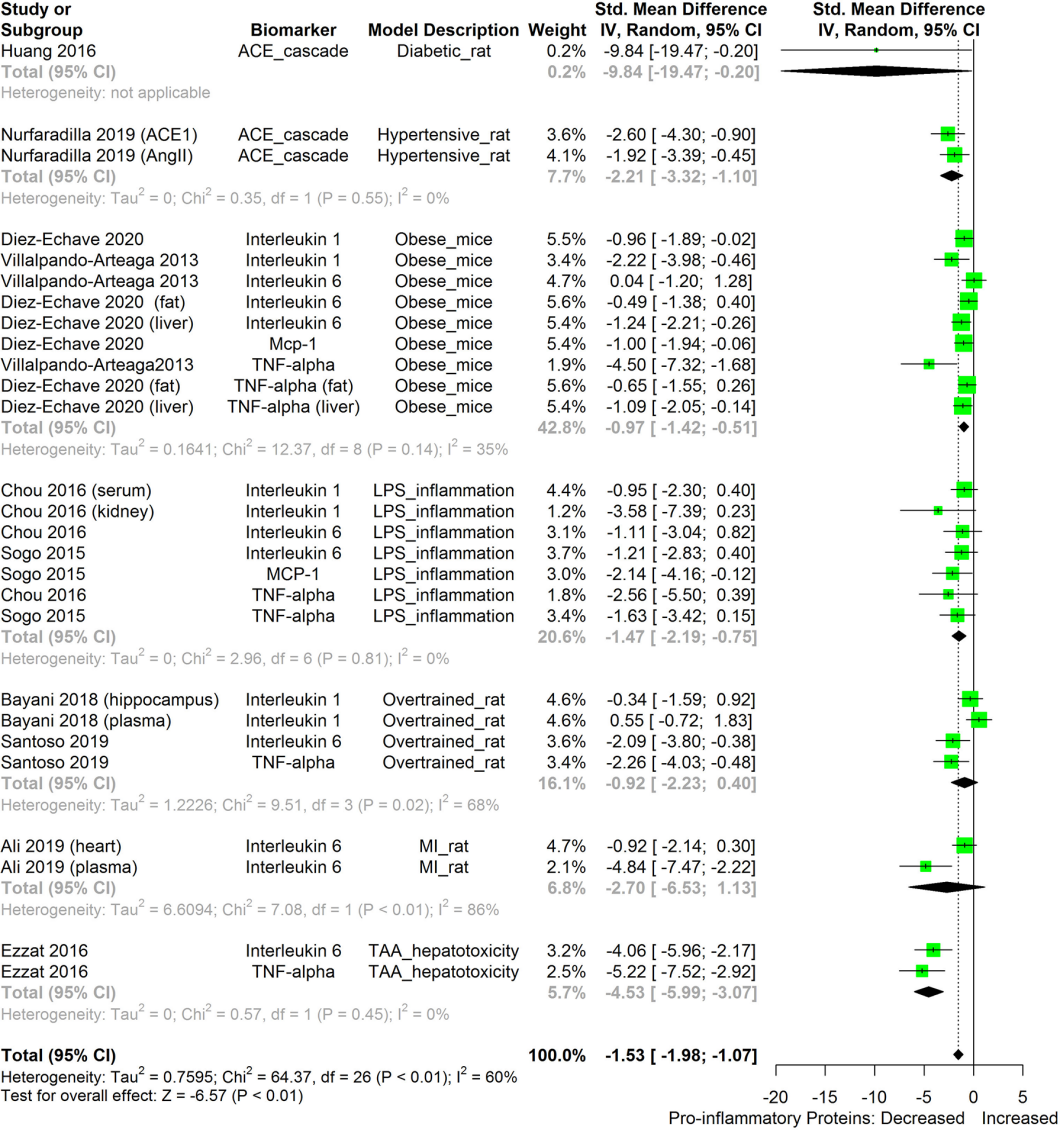
Meta-analysis and forest plot of the effect of HS administration on selected pro-inflammatory biomarkers (ACE, TNF-α, IL-6, IL-1β, MCP-1) across different diseased-rodent models. The meta-analysis was sub-grouped by rodent’s disease models (i.e diabetic rats, hypertensive rats, LPS-inflammation model, over-trained rats, MI-rats, TAA-hepatotoxicity models). The standardized mean difference (SMD), which is an estimate of the effect size, is presented as a square on the forest plot, for each study and as a diamond for the pooled estimate of each subgroup or overall studies. The overall pooled SMD for all included studies is shown in the bottom section of the forest plot. SMD data points that are lower than zero signify that treatments with HS lowered the level of the biomarker while those greater than zero signify that HS treatment causes an increase in the levels of the biomarker. The statistical significance of the pooled estimate is indicated by the Z test of significance. MI, Myocardiac infarction; LPS, Lipopolysaccharide; TAA, Thioacetamide; TNF-alpha, Tumor necrosis factor-alpha; MCP-1, Monocyte chemoattractant protein-1; ACE, Angiotensin-converting enzyme.

We also attempted to compare the immunosuppressive potential of HS in different animal disease models using TNF-α, IL-6, IL-1β, MCP-1, and ACE as biomarkers (**Figure 13**). The levels of the inflammatory biomarkers TNF-α, IL-6, IL-1β, MCP-1, and ACE appeared to be more readily suppressed by HS in some disease models than in other disease models. HS appears to have the lowest effect in obese mice; however, the low number of studies on the subject limits the strength of this line of reasoning.

### Effects Across Different Experimental Models

The anti-inflammatory effects of HS calyx extract were observed in healthy mice (41), healthy humans (20, 47), and in different disease models such as dextran sodium sulfate-induced colitis mice (40), xylene-induced ear edema mice (1), sucrose induced metabolic syndrome rat (45), hypertensive rat (two-kidney-one-clip (2K1C) model of hypertension (26), men with low risk of cardiovascular-disease (51), human subjects with metabolic syndrome (42, 50), Dimethylbenz(A)Anthracene (DMBA) treated rats (39), high fat diet (HFD) induced obese mice (9, 16, 46), STZ-induced Diabetic rats (37, 46), LPS-induced inflammation in rats (38), hypertensive humans (24), myocardial infarction rat (44), LPS-induced inflammation in mice (11, 48), over trained rats (43, 52), N(G)-nito-L-arginine-methyl ester (L-NAME)- induced hypertensive rats (25), Thioacetamide (TAA)-induced hepatotoxic rats (17) and 12-O-tetradecanoylphorbol-13-acetate (TPA) induced mouse ear edema (49) as shown in **Table 2**.

**Table 2:** HS effects on inflammation biomarkers in included studies.

S/N	First Author, year	Experimental Model	HS formulation	Dosage/route	Inflammatory mediator investigated	Tissue Examined	Sample size (n) (*)	Methodological Instrumentation	Major findings	Reference
1	Fakeye, (2008)	Healthy Mice	Aqueous and ethanol Extract of HS	300 mg/kg body weight, oral administration for 28 days	IL-10, TNF- α	Serum	10	ELISA	IL-10 (-) (#3)	(41)
									TNF- α (↓)(#3)	
2	Nurkhasanah (2015)	DMBA treated rats ()	Ethanol extract	10 (mg/kg body weight) oral administration for 21 days before toxicity induction with DMBA	IL-10, IL-14	Spleen	not stated	immunohistochemistry	IL-10 (↑)	(39)
									IL-14 (↑)	
3	Villalpando-Arteaga (2013)	High fat diet induced obese mice	Aqueous Extract (room temperature)	33 mg anthocyanin equivalent per kg. Administration of HS was done thrice weekly for 8 weeks using an orogastric-way.	IL-1β, IL-6, IL-10, TNF- α	Liver	10	PCR	IL-6 (-)	(9)
									IL-10 (-)	
									TNF- α (↓)	
									IL-1β (↓)	
4	Wang (2011)	STZ-induced Diabetic rats	Aqueous Extract 100°C	100 mg/kg body weight, fed orally to the animals for 8 weeks after which diabetes was induced by STZ injection	NF-κB	Liver	10	Electrophoretic Mobility Shift Assay (EMSA)	Up regulated expression of NF-κB	(37)
5	Kao (2009)	Rats challenged with LPS	Methanol/ethyl acetate extract	20 mg/kg body weight oral administration for 5 days.	Cyclooxygenase-2	Liver	10	Western blotting/Chemiluminescence	Down regulated Cyclooxygenase-2 expression	(38)
6	BeltranDeb´on (2010)	Healthy Human	Aqueous Extract (100°C)	10 g administered orally 3 hours before study.	IL-8, IL-6, MCP-1	Blood Plasma	10	multiplex cytometric bead-based assay	IL-6 (nd)	(20)
									IL-8 (nd)	
									MCP-1 (↓)	
7	Beltran-Deb´on (2015)	Healthy Human	Aqueous Extract	5g of extract (569 mg of polyphenol) was administered orally for 3 hours before-study	IL-6, IL-8, CRP protein, ACE, TNF-α	Blood Serum	10	ELISA	IL6 (-) (#1)	(47)
								hs-CRP kit from Biokit.	IL8 (-) (#1)	
									CRP (-) (#1)	
									ACE (↓) (#1)	
8	Herrera-Arelano (2007)	Hypertensive Human	Aqueous extract (60°C)	250 mg anthocyanin administered orally for 4 weeks.	ACE	serum	171	HPLC/Chromolite Column	ACE (↓)	(24)
9	Ali (2019)	Rat model of myocardial infarction	Aqueous extract	100 mg/kg body weight administered orally for 7days	IL-6, IL-10	Plasma/Heart	12	ELISA	IL-6 (↓)	(44)
									IL 10 (↓)	
10	Bayani (2018)	Over trained Rat	Ethanol Extract	500 mg/kg body weight administered orally shortly before overtraining for 11 weeks	IL-1β	Plasma, Hippocampus	10	ELISA	IL-1β Plasma (-)	(52)
									IL-1β Hippocampus (-)	
11	Sogo (2015)	LPS-induced inflammation in Mouse	Delphinidin	Intraperitoneal administration of 15 µMl/kg body weight for 4 days before LPS treatment	TNF-α, IL-6, MCP-1, Paw edema	Serum, paw	8	ELISA	TNF-α (↓)	(11)
			3-sambubioside and Delphinidin						IL-6 (↓)	
			Purified from HS						MCP-1 (↓)	
									Paw edema (↓)	
12	Chou (2016)	LPS-induced inflammation in Mouse	Aqueous extract (4°C)	200 mg/kg body weight orally administered for 7days before LPS treatment	Il12b,Il15,Il17ra,Il17c,Il1β,Il1rap,Il6,Il22,Ifitm10, Ifi47,Ifnb1,Igtp,Iigp1,Irf1,Irf8, Ccrl2 Cxcl11,Cxcl2,Cxcl9,Ccl12,Ccl22,TNF-α, Tnfsf10, Tnfsf15,Cox-2	Kidney	10	Microarray	Il12b (↓)	(31)
									Il15 (↓),	
									Il17ra (↓)	
									Il17c (↓)	
									Il1β (↓)(#2)	
									Il1rap (↓)	
									IL-6 (↓)(#2)	
									IL-22 (↓)	
									Ifitm10 (↓)	
									Ifi47 (↓)	
									Ifnb1 (↓)	
									Igtp (↓)	
									Iigp1 (↓)	
									Irf1 (↓),	
									Irf8 (↓)	
									Ccrl2 (↓)	
									Cxcl11 (↓)	
									Cxcl2 (↓)	
									Cxcl9 (↓)	
									Ccl12 (↓)	
									Ccl22 (↓)	
									TNF-α (↓)(#2)	
									Tnfsf10 (↓)	
									Tnfsf15 (↓)	
									Cox-2 (↓)	
									iNOS2 (↓)(#2)	
									NF-κB (P56) (↓)	
13	Abdel-Rahman (2017)	L-NAME- induced hypertensive rats	Hydro-alcoholic extract of *Hibiscus*	250 mg/kg body weight administered orally alongside with L-NAME for 4weeks.	ACE	Blood serum	14	ELISA kit	ACE (↓)	(25)
			*sabdariffa* and *Oleaeuropaea* in a ratio 2:1 respectively.							
14	Ezzat (2016)	TAA-induced hepatotoxic rats	Trifluoroacetic acid extract of HS	100 mg/kg body weight administered orally for 5 days per week for four weeks.	TNF-α, IL-6, IFN-γ, NF-κB	Blood serum	16	ELISA kit	TNF-α (↓)	(17)
									IL-6 (↓)	
									IFN-γ (↓)	
									NF-κB (↓)	
15	Asgary (2016)	Human subject with metabolic syndrome	Encapsulated powdered HS	500 mg of encapsulate HS (3 mg anthocyanin equivalent) oral administration for 4weeks.	hs-CRP	Serum	113	Not stated	hs-CRP (-)	(50)
16	Joven (2014)	Human subjects with metabolic syndrome	Polyphenol Extract from HS	125 mg/kg of HSE was administered orally twice daily for 4 weeks	2-microglobulin,ACE,CCL2, hsCRP, IL-1β, IL-6, IL-8, TNF-α	Serum	31	ELISA, AutoAnalyser	CRP (–) (#1)	(42)
									IL-6 (↓) (#1)	
									ACE (-) (#1)	
									CCL2 (↓) (#1)	
									IL-1β (↓) (#1)	
									IL-8 (↓) (#1)	
									TNF-α (↓) (#1)	
									2-microglobulin (-) (#1)	
17	Abubakar (2019)	Men with low risk of cardiovascular-disease	Aqueous extract (100°C)	One time oral administration of 7.5 g of HS extract (containing 150 mg total anthocyanins and 311mg gallic acid)	CRP	plasma	22	AutoAnalyser	CRP (-)	(51)
18	Santoso (2019)	Over-trained Wistar Rat	Methanolic calyx extract	500 mg/kg body weight administered orally shortly before overtraining for 11 weeks	IL-6, TNF-α	Heart	10	ELISA	TNF-α (↓)	(43)
									IL-6 (↓)	
19	Nurfaradilla (2019)	Hypertensive Rat (2K1C model of hypertension)	Aqueous Extract (50°C)	150 mg/Kg body weight, administered orally for two weeks	Serum ACE, Plasma Ang II.	Plasma/ serum	12	Spectrophotometry UV-Vis, ELISA	ACE (↓)	(26)
									Angiotensin II (↓)	
20	Huang (2016)	HFD and STZ-induced diabetic rat model	Polyphenolic extract from HS	200 mg/kg body weight was orally administered along side HFD for seven weeks before STZ injection	AT1 receptor	Kidney	16	Radioimmunoprecipitation assay	AT1 receptor (↓)	(46)
21	Diez-Echave (2020)	HFD-induced obesity mice	Aqueous extract of HS	Oral administration for 42 days at 1, 10, 25 mg/kg body weight	TNF-alpha, IL-1β, IL-6, MCP-1, TLR4	Liver Adipose	20	RT-qPCR	TNF-alpha (↓)	(16)
									IL-1β (↓)	
									IL-6 (↓)	
									MCP-1 (↓)	
									TLR4 (↓)	
22	Perez-Torres (2013)	Sucrose induced Metabolic syndrome rat model	Aqueous Extract (100°C)	Unclear dosage and route, 24 weeks treatment.	iNOS	Liver	14	immunoblotting	iNOS (↓)	(45)
23	Tseng (1998)	TPA induced mouse ear edema model	Protocatechuic acid extract of HS	10 or 20umol applied on the skin twice daily for 4 days.	Ear edema	Ear	15	Gravimetric	Ear edema (↓)	(49)
24	Nwachukwu (2015)	Mild to moderate essential hypertensive humans	Aqueous Extract (100°C)	Oral administration of 150mg/kg body weight/day, for 4 weeks	ACE	Serum	50	HPLC	ACE(-)	(14)
25	Dafallah (1996)	Carrageenan induced paw edema rat model	Aqueous Extract (room temperature)	500 mg/kg body weight orally administered 3 hours before carrageenan injection	Paw Edema	Paw	12	Water Displacement method	Paw Edema (-)	(53)
26	Lubis (2020)	dextran sodium sulfate-induced colitis mice model	Ethanol extract	300 mg/kg body weight/day administered orally for 28 days	IL-6 and TNF-α, IL-10, HCIS.	Colon	11	Histopathology and Immunohistochemistry	IL-6 (↓)	(40)
									TNF-α (↓)	
									IL-10 (↑)	
									HCIS (↓)	
27	Ali (2011)	Xylene-induced ear edema mice model	Ethanol extract	A one-time oral HS administration at 250 and 500 mg/kg body weight	Ear edema	Ear	15	Gravimetric	Ear edema (↓)	(1)

(*) sample size used for control and test group.

(nd) not detected.

(↓) shows significant decrease of inflammatory mediator.

(↑) shows significant increase of the inflammatory mediator.

(-) shows insignificant difference between the treated and untreated groups.

(#1) The SD value used for meta-analysis was estimated using Revman5.3.

(#2) The data reported in **Table 2** of Chou et al. (31) was transformed for meta-analysis using the formula below, EU=(LPS+1), ET=(EU+LPSroselle). where EU is the transformed data for the exposed-untreated group, LPS is the data reported for LPS exposed, and LPSroselle is the data reported for the treated group. ET is the transformed data for the exposed and treated group.

(#3) The individual outcomes reported in **Tables 1** and **2** of Fakeye et al. (41) were used to generate a mean and SD value that was used for the meta-analysis.

2K1C, Two-kidney-one-clip; ACE, Angiotensin-converting enzyme; Ang II, Angiotensin II; AT1 receptor, Angiotensin II type 1 receptor; Colitis Inflammation Score; CRP, C-reactive protein; DMBA, Dimethylbenz(A)Anthracene; ELISA, Enzyme-linked immunosorbent assay; HCIS, Histological; HFD, High fat diet; HPLC, High Performance Liquid Chromatography; HS, Hibiscus sabdariffa; hs-CRP, High-sensitivity C-reactive protein; IFN-γ, Interferon-γ; IL-10, Interleukin 10; IL-14, Interleukin 14; IL-1β, Interleukin 1β; IL-6, Interleukin 6; IL-8, Interleukin 8; iNOS, inducible Nitric Oxide synthase; L-NAME, N(G)-nito-L-arginine-methyl ester; LPS, Lipopolysaccharides; MCP-1/CCL2, Monocyte Chemoattractant Protein-1; NF-κB, nuclear factor kappa B; PCR, Polymerase chain reaction; RT-qPCR, Quantitative reverse transcription PCR; STZ, Streptozotocin; TAA, Thioacetamide; TLR4, Toll-like receptor 4; TNF-α, Tumor necrosis factor; TPA, 12-O-tetradecanoylphorbol-13-acetate.

Significant alterations were observed in the levels of most of the inflammatory biomarkers investigated following induction of each disease condition when compared to their respective un-induced controls. HS extract exerted significant anti-inflammatory effects against most of the investigated biomarkers of inflammation. With a few exceptions, the levels of most of the investigated inflammatory cytokines in healthy models were generally low, sometimes undetectable (20), and often without significant alterations after treatment with HS. Some exceptions included the suppression of TNF-α in healthy mice (41), suppression of MCP-1, and ACE1 in healthy humans (20, 47). Significant changes were not observed in the levels of CRP following treatments with HS in healthy humans (20), human subjects with metabolic syndrome (50), and men with low risk of cardiovascular disease (51).

#### Extract Formulations

A description of the extract formulation of each included study is presented in **Table 2**. Anti-inflammatory activities were observed in different formulations of HS calyx such as ethanol extract (1, 25, 40, 41, 52), hot or cold aqueous extract (9, 16, 20, 24, 31, 37, 45, 51), methanol extract (38, 43), trifluoroacetic acid (TFA) extract (17), Polyphenol Extract (42), and protocatechuic acid extracts (49) of HS calyces. A study done with the encapsulated whole calyx powder did not yield a significant difference in the level of CRP when compared with the untreated group.

#### Affected Organs

The tissues/organs that were analyzed in each study are presented in **Table 2**. HS calyx extract elicited significant anti-inflammatory effects in different tissues and organs including blood (11, 17, 24, 25, 41, 47); spleen (39); liver (9, 37, 38, 45); heart (42–44, 50); kidney (31, 46), liver Adipose (16); ear (1, 49); colon (40), and paw (11). Significant alteration was not detected in IL-1β levels in the hippocampus of rats following treatment with HS calyx extract (52).

The route of administration, the applied doses of HS extract, and the duration of treatment in the included studies are shown in **Table 2**. All but two studies administered HS extract formulation orally. Sogo et al. (11), administered pure anthocyanin from HS intraperitoneally while Tseng et al. (49) applied protocatechuic acid extract of HS topically. In most of the studies (with few exceptions), the administration of HS calyx extract on experimental subjects lasted up to (or more than) three weeks. However, HS was administered for three hours in the works of Beltrán-Debón et al. (20), Beltrán-Debón et al. (47), and Dafallah et al. (53). HS administration lasted between four to 7 days in the works of Sogo et al. (11), Tseng et al. (49), Kao et al. (38), Chou et al. (31), and Ali et al. (44). HS treatment lasted for two weeks in the study of Nurfaradilla (26). One time administration of HS was reported in the work of Abubakar et al. (51) and Ali (1).

## Discussion

### Chemical Constituents and Bioactivity of Different HS Extract Formulations

The anti-inflammatory effect of HS extracts has been reported in many studies using various formulations of HS. Each of these formulations contains different proportions of bioactive agents which contribute to the observed bioactivities. The aqueous extracts of HS have comparatively higher contents of cyanidin-3-sambubioside and delphinidin-3-sambubioside as well as a higher antioxidant activity than methanol, ethylacetate, and formic acid extract (2). Flavonoids are more enriched in extracts obtained using less polar solvents (6). Hydroxycitric, hibiscus, and chlorogenic acid are the most abundant phenolic acids detected in aqueous extracts of HS (50). Delphinidin 3-sambubioside and Cyanidin 3-sambubioside are the predominant anthocyanins present in aqueous extract of HS (9, 26, 31, 50).

The mechanisms involved in the anti-inflammatory activities of HS extract appear to be multifunctional, involving different bioactive agents which can interact with different biological targets to elicit the observed anti-inflammatory effects. Immunomodulatory activities have been reported for pectin-rich polysaccharide obtained from water soluble fractions of HS (4). HS extracts containing PCA (7%), catechins (9.97%), epigallocatechin (10.23%), epigallocatechin-3-gallate (20%), and caffeic acid (18.%) have been shown to possess in-vitro antioxidant, nitric oxide inhibitory and Prostaglandin E2 inhibitory activities (38). The antioxidant capability was improved in diabetic rats after the intake of PCA and gallic acids extracted from HS (21). Metabolites of Delphinidin-3-glucoside have strong radical scavenging activities (11). Essential oils obtained from HS (containing fatty acids such as hexadecanoic and 14-methyl-pentadecanoic acid methyl ester) were reported to elicit anti-inflammatory activity against LPS-stimulated RAW264.7 cells (18). Anthocyanin-rich water soluble extracts of HS inhibited ACE1 enzyme in-vitro (12). Delphinidin-3-sambubioside containing extract of HS (obtained using ethylacetate and methanol) has been reported to possess cyclooxygenase 1 inhibitory activity in-vitro (8).

### Bioavailability and Pharmacokinetics of HS Extracts Formulation

HS is most commonly consumed as a decoction of the dried HS calyx. Many in-vivo and in-vitro studies have shown the immunosuppressive effect of aqueous HS extract. To fully elucidate the mechanism of the anti-inflammatory activities of HS, the bioavailability of the bioactive constituents of HS need to be considered. Within 1 hour after ingesting aqueous extract of HS, a small percentage (1%) of its flavonoids and anthocyanin contents are rapidly absorbed and metabolized before appearing in the blood plasma as flavonoid conjugates and hippuric acid respectively (10, 51). Anthocyanin glycosides are more readily absorbed than the aglycone equivalent (11). Flavonoid O-glucosides are more readily absorbed than their aglycone form or derivatives with rhamnose glycosylation (54). Urinary levels of the metabolites of anthocyanin peak around 2 hours following the intake of HS extract (5). Unabsorbed anthocyanin is further metabolized by intestinal microbiota which hydrolyses and stimulates the absorption of the aglycones of anthocyanin (11). Unabsorbed anthocyanin is excreted through the feces (10). PCA is the main metabolite found in rat’s plasma after the consumption of cyanidin 3-O-glucoside (11, 55). Less than 1% of the ingested phenols were detected in the plasma, with plasma levels peaking within 90 minutes of HS intake (51). The hibiscus acid content of HS extract is metabolized into hydroxycitric acid, which attains its peak plasma level in about 2 hours following ingestion (54). The presence of chlorogenic acid, methyl digallates, and feruloyltyramine in the plasma within 1 hr of HS ingestion suggests the possibility of direct absorption of the phenolic acids into the bloodstream in un-metabolized forms (54). Abubakar et al. (51), also reported the detection of 3-O-methylgallic acid, hippuric acid, gallic acid, and 4-O-methylgallic acid in blood plasma following the intake of HS extract. Fernandez-Arroyo et al. (54), report that about 5 µM of flavonols were detected in blood plasma within 2 hours of intake of 1200 mg/kg of HS polyphenol extract. The foregoing suggests that many components of HS have low bioavailability and are metabolically transformed and eliminated through urine within about 2 hours of ingestion.

### Potential Mechanisms of the Immunosuppressive Activity of HS Extract

As discussed previously, the presence of different bioactive agents in extracts of HS, suggests the possible involvement of several mechanisms in the suppression of immune and inflammatory responses. Suggested mechanisms include the suppression of cellular stress, inhibition of enzymes that can trigger inflammatory reactions, and down-regulation of pro-inflammatory genes.

#### Suppression of Cellular Stress

An important mechanism involved in the anti-inflammatory activities of HS is its ability to suppress the generation of oxidative stress and cellular damage in cells. Several pathological conditions are associated with oxidative stress. Increased oxidative stress can cause cellular damages leading to the release of stress factors such as heat shock protein, reactive oxygen species (ROS), necrotic, and apoptotic factors, etc. These factors are capable of activating TLR proteins in immune cells leading to downstream expression of several inflammatory genes (56). Oxidative stress is reported in most of the different experimental disease models used in the included studies. For instance LPS treatment depleted the anti-oxidant status of cells (38), over-training elevated the levels of malondialdehyde (57), models of hypertension had elevated malondialdehyde levels as well as depleted glutathione (25), mechanism of Isoprenaline -induced myocardiac infarction involves free radical generation, increased necrosis, inflammation and cardiac fibrosis (44), TAA-intoxication causes an increase in reactive oxygen species (ROS) which may result in centrilobular necrosis (17), metabolic syndrome and non-communicable diseases such as diabetes and obesity can be caused by excess oxidative stress arising from disrupted cellular redox control (58).

Several components of HS extract have been demonstrated to possess high radical scavenging potentials in in-vivo and in-vitro studies. Anthocyanins can reduce oxidative stress through the direct scavenging of free radicals or the induction of anti-oxidant enzymes (10). HS possesses potent in-vitro anti-oxidant capability (2). Anti-oxidant capacity was improved in diabetic rats after the administration of PCA and gallic acids extracted from HS (21). The phenolic acid constituents of HS, though short-lived in the plasma, can improve the anti-oxidant capability of the cells in a mechanism that involves iron homeostasis (54, 59). In mechanisms that involve the enhancement of antioxidant capabilities, anthocyanin has been reported to trigger the activation of protein kinase B signaling, inhibition of inhibitor of κB phosphorylation, and the suppression of NF-κB in hydrogen peroxide (H_2_O_2)_-stimulated rat dermal fibroblast (60). The conjugated forms of quercetin and kaempferol often detected in the plasma after intake of HS could indicate long-lasting cellular antioxidant effects because of their long plasma half life (54). Some of the anti-inflammatory properties of anthocyanin may be explained by the antioxidant effects they exhibit (10). Beltrán-Debón et al. (47) suggest the possible involvement of the Heme-oxygenase (HO)-biliverdin reductase system in the in-vivo antioxidant and anti-inflammatory effect of HS. When the anti-oxidant capacity of cells are improved following HS treatments, the levels of oxidative stress-induced damages would be suppressed and TLR stress related agonist will not be generated.

#### Inhibition of Enzymes Capable of Triggering Immune Responses

##### ACE

Direct Inhibition of enzyme systems that are capable of stimulating inflammatory response can explain some of the anti-inflammatory activities of HS. One of such enzyme systems is the ACE1. ACE1 is a component of the Renin-Angiotensin-System which functions in the homeostatic control of pressure in arteries, perfusion of tissues, and regulation of extracellular cell volume. The Renin-Angiotensin-System system consists of two ACE enzymes, ACE1 and angiotensin converting enzyme -2 (ACE2), which functions in an opposing manner to both increase and decrease vasoconstriction respectively (61). ACE1 catalyzes the conversion of Ang I into Ang II which then activates the AT1 receptor to trigger several downstream processes including vasoconstriction, sodium/water re-absorption, proliferation and hypertrophy, fibrosis, oxidative stress, and arrhythmogenesis (61). Ang II can promote vascular permeability, leukocyte infiltration, activate the Nf-κB signaling pathway, and inadvertently produces inflammatory mediators (25). HS administration has been reported to suppress both Ang II and AT 1 receptors in diabetic and hypertensive rat models (26, 46). In-vitro studies have also demonstrated the ACE1 inhibitory effects of Anthocyanin-rich extracts of HS (12). The inhibition of ACE1, Ang II, and AT 1 receptor by HS extract could potentially inhibit Ang II-mediated activation of the AT1 receptor. This would subsequently suppress the downstream activation of Nf-κB as well as ROS production (30).

ACE2, a homologue and regulatory arm of ACE 1, diverts some of the Ang II generated by ACE1 into the production of a potent vasodilatory growth inhibitory peptide, angiotensin (1–7), which activates the Mas AT2 receptor and receptor Mas (61). Activation of the AT2 receptor is thought to enhance ACE2 activities and subsequently leads to an ACE2-mediated inhibition of NF-kB pro-inflammatory signaling as well as the downstream inhibition of TNF-α-mediated ICAM-1 expression (62). ACE2 knockout mice were observed to suffer from more pulmonary inflammation following exposure to cigarette smoke (63), probably as a result of unregulated ACE1 activity. Conditions that suppress the level of ACE2, for instance as observed in coronavirus binding to ACE2 (64, 65) would interrupt the immunomodulatory activities of ACE2 thereby bringing about an increase in AT1 receptor-mediated pro-inflammatory responses (66). Agents capable of inhibiting ACE1 activities hold promise in the management of pulmonary inflammatory disorders (66).

Inhibition of components of the angiotensin converting enzyme (ACE) cascades by HS extract have been reported in both human and animal studies (24, 25). The result obtained following the meta-analysis of the effects of HS on components of the ACE cascade, as shown in this review (**Figure 3**), showed that HS administration had an overall inhibitory effect on the ACE cascade. Possible mechanisms of the HS-induced inhibition of components of the ACE cascade may involve both direct and indirect mechanisms. Delphinidin-3-O-sambubioside and cyanidin-3-O-sambubioside from HS have been reported to competitively inhibit ACE activity in-vitro (13). Polyphenols from HS were observed to suppress the expression of AT1 and AT2 receptors in HK-2 cells that were exposed to high glucose but did not have a significant effect on the expression of the AT1 and AT2 receptors in HK-2 cells that were not exposed to high glucose (15). The inhibition of the ACE cascade would possibly suppress the conversion of Ang 1 to Ang II thereby halting the downstream consequences of AT1 receptor activation and the consequent production of oxidative stress and inflammatory responses (25).

##### Lipoxygenase and Cyclooxygenase

HS has also been reported to inhibit lipoxygenase and cyclooxygenase enzyme systems (10, 59). This enzyme functions in the conversion of arachidonic acid to prostaglandins. The over expression of the enzymes of prostaglandin synthetic pathways (COX-2 and lipooxygenase), can lead to different pathologies such as renal disorders and nephritis (31). HS treatments significantly lowered the activities of COX-2 in-vivo studies (31). Lipoxygenase was inhibited in-vitro by PCA from HS extract (59). This implies that treatment with HS could lower the level of prostaglandin and suppress prostaglandin-mediated inflammatory responses. Prostaglandin E2 has been reported to contribute to the formation of pulmonary edema (67). The observed reduction in edema formation (**Figure 4**) following treatment with HS may be explained in part by the inhibition of the enzymes of the prostaglandin synthesis by HS.

#### Down-Regulation of Pro-Inflammatory Genes

Down-regulation of pro-inflammatory genes and factors is another potential mechanism involved in HS anti-inflammatory activities. Different in-vivo and in-vitro studies have demonstrated the ability of HS or known components of HS to downregulate the expression of pro-inflammatory genes such as NF-κB, TNF-α, interleukins, iNOS (17, 20, 31, 44). The exact mechanism involved in the down-regulation of pro-inflammatory genes are unknown but accumulating evidence suggests that HS may affect one or more upstream component of the pro-inflammatory gene expression cascade. An instance is the HS-induced suppression of the levels of Ang II and AT1 receptors (26, 46) which prevents the downstream NF-κB mediated expression of the pro-inflammatory gene. There is also the possibility that there is post transcriptional modulation of inflammatory gene products. Ali et al. (44) reported that IL-6 protein levels were significantly reduced by HS whereas IL-6 (mRNA) was not significantly affected in a myocardiac infarction model. There is also the possibility of direct interaction between components of HS extract and the surface receptors of immune cells that can lead to modulation in gene expression. For instance, Zheng et al. (23), reported that polysaccharide extract of HS increased proliferation and NF-κB expression of RAW264.7 cells in a mechanism that was likely due to the enhancement of macrophage proliferation or the activation of specific surface receptors on macrophages. The anti-inflammatory activity of Delphinidin 3-sambubioside and Delphinidin against LPS-induced cells were reported to be independent of direct TLR4 binding interaction (11).

Microarray gene profiling studies by Chou et al. (31), showed that 26 genes including interleukins, interferons chemokines, tumor necrosis family, as well as COX and iNOS genes were significantly altered following LPS-intoxication and subsequent HS treatment in mice kidney (31). Polyphenol extracts from HS inhibited the activation of c-Jun N-terminal kinase (JNK) and p38 mitogen-activated protein kinase (MAPK or MAP kinase) in LPS-challenged rats hereby suppressing NF-κB activation and the downstream expression of COX-2 proteins (38). The metabolites of protocatechuic acid and cyaninin can suppress COX-2 and iNOS expression (10).

#### NF-κB

Treatment with HS extract has been reported to down-regulate the activity of NF-κB (p65) subunit in different animal and cell line models (17, 18, 31). NF-κB is a family of inducible transcription factors that regulate several genes which function in inflammation and immune responses (68). NF-κB is ubiquitously expressed and comprises several subunits such as NF-κB1 (p50), NF-κB2(p52), RelA(p65), RelB, and c-Rel (69). NF-κB is commonly associated with an inhibitory subunit, IκB. NF-κB can be activated in a pathway that is either dependent or independent on the IκB complex degradation. NF-κB is typically activated through the degradation of its associated IκB-α upon phosphorylation by the IKK complex. The IKK itself is activated by several upstream signals including cytokines, growth factors, antigenic factors (66, 70). On activation, NF-κB can migrate into the nucleus to activate gene expression (71). Dysfunctional NF-κB signaling has been implicated in several inflammatory disorders (68). Suppression of NF-κB activity will reduce the downstream expression of pro-inflammatory genes thereby effectively attenuating immune responses (18).

#### IL-1β

The results from the meta-analysis suggest that HS administration suppressed the levels of IL-1β across different experimental models (**Figure 6**). IL-1β and TLRs are membrane bound proteins that are commonly expressed on immune cells. TLRs are activated by the binding of antigenic substances from bacteria and viruses or stress signals from the host (72). IL-1β is a component of the innate immune response that is rapidly produced following stimulation of TLR or interleukin-1 receptor (IL-1R) by microbial antigenic substances or stress signals from the host (Martin2002, Thobakgale2017). IL-1β also facilitates the maturation, proliferation, and differentiation of immune cells (73). IL-1R is activated by IL-1β. Following the binding of its agonist, IL-1R triggers the release of inflammasomes which matures into active IL-1β and then mediates inflammatory reactions (74). Activation of IL-1R and TLR triggers a cascade of signaling which involves the interleukin-1 receptor associated kinase (IRAK)/MAPK/JNK2/extracellular signal-regulated kinases (ERK) and eventually culminates in the activation of NF-κB and other transcription factors that are involved in the expression of inflammatory mediators such as MCP-1, IL-8, and IL-6 (74). Hyper-activation or dysfunctional regulation of the activities of the IL-1R and TLR can cause tissue damage and auto-immune diseases (74).

#### TNF-α

The result from the meta-analysis indicates that HS supplementation significantly suppressed the levels of TNF-α across the different studies (**Figure 8**). TNF-α are mainly produced by immune cells such as activated macrophages, lymphocytes, endothelial, epithelia cells, smooth muscle cells, and cardiac myocytes (71). It participates in pro-inflammatory reactions. TNF-α, on binding to its receptors (Tumor necrosis factor receptor (TNFR) 1 and 2) triggers the activation of NF-κB, MAPK/c-Jun, and caspase apoptotic signaling in a process mediated by the TNF receptor associated factor 2 (TRAF2) (71). The activated Mitogen Activated Protein (MAP) kinase kinase kinase (MAP3K)/JNK system generates c-Jun and Activation protein 1 (AP-1) factors that activate the expression of pro-inflammatory genes (71). The persistent overproduction of TNF-α could be deleterious to tissues (71). The ability of HS to inhibit TNF-α-induced activation of NF-κB presents an interesting prospect for the use of HS in managing inflammatory disorders. Inhibition of TNF-α reduces the upstream signals that can potentially stimulate the NF-κB induced expression of inflammatory cytokines.

#### MCP-1

Evidence from this meta-analysis shows that HS treatment can significantly suppress the levels of MCP-1 (**Figure 2**). Furthermore, the low heterogeneity (0%) observed following the meta-analysis of the evidence gathered from both human and animal studies, suggests that the MCP-1-lowering effect of HS is independent of the type and health state of the model. MCP-1 are inflammatory chemokines that regulate the migration and infiltration of macrophages and monocytes to sites of inflammation (75). The levels of MCP-1/CCL2 and its receptor C-C chemokine receptor type 2 (CCR2), are increased in different inflammatory related diseases such as atherosclerosis, inflammatory bowel disease, allergic asthma, rheumatoid arthritis, and Type 2 diabetes, etc. (75). The increased expression of MCP-1 in the kidney and vasculatures in hypertensive subjects results in increased infiltration of monocytes expressing CCL2 receptors (CCR2) into the kidney (76). The inhibitors of MCP-1 could lower excessive renal accumulation of monocytes and macrophages (77). While many inflammatory cytokines are either undetectable or very low in a healthy subject, It is interesting to note that MCP-1 was present in healthy subjects and was significantly lowered after HS treatment (20). The mechanism involved in HS-induced reduction in MCP-1 levels does not involve the enhancement of the antioxidant capability of the plasma (20). The ability to suppress MCP-1 levels is one of the prominent anti-inflammatory potentials of HS. Since most of the inflammatory cytokines are secreted by specialized immune cells, suppression of MCP-1 activity would reduce the aggregation of circulating monocytes at inflammation sites. This would reduce the numbers of circulating monocytes that are able to develop into specialized cytokine-releasing immune cells and thus reduce the consequent inflammation to body tissues.

#### IL-6

The meta-analysis showed that HS administration significantly suppressed the cellular levels of IL-6 in different disease models (**Figure 6**). IL-6 are pro-inflammatory cytokines that are produced in response to the presence of inflammatory stimuli such as IL-1, LPS, TNF-α, and IL-4 (78). IL-6 acts by binding to soluble or bound IL-6 receptors which are expressed on many endothelial cells. The downstream effect of IL-6 includes increased expression of the AT1 receptor (78), adhesion molecules [including ICAM-1, vascular cell adhesion molecule 1 (VCAM-1)], vascular endothelial growth factor (VEGF), and other inflammatory cytokines such as MCP, IL-8, and IL-6 (79, 80). Dysfunctional IL-6 signaling has been implicated in autoimmune diseases, chronic inflammation endothelial. dysfunction, and fibrogenesis. Suppressing IL-6 signaling could be beneficial to patients with these disease conditions (81). In many disease models, inhibition of IL-6 expression has been shown to prevent, heal or suppress disease progression (82). The IL-6 suppressing effect of HS across different disease models suggests a role for HS in the management of chronic inflammatory diseases.

#### IL-10

IL-10 is an anti-inflammatory cytokine produced by virtually all immune cells. Its main function is to prevent immunopathology during inflammatory responses (83). The result obtained from the pooled meta-analysis suggests that IL-10 was not significantly altered by HS treatment (**Figure 10**). This could, however, be due to the small number of studies used for the meta-analysis.

#### iNOS

Inducible nitric oxide synthase (iNOS) synthesizes nitric oxide a regulator of immune responses. Overexpression of iNOS and the consequent over production of nitric oxide can lead to cellular injury including DNA damage (31). Findings from studies conducted by Chou et al. (31) and Perez-Torres et al. (45) suggest that HS can lower iNOS. This makes HS potentially useful in protecting against nitric oxide-induced cellular damage in the conditions of chronic inflammatory diseases. Our meta-analysis of the available evidence (**Figure 11**) showed that HS lowered iNOS, however, the result was not statistically significant (p=0.14).

**Figure 14:**
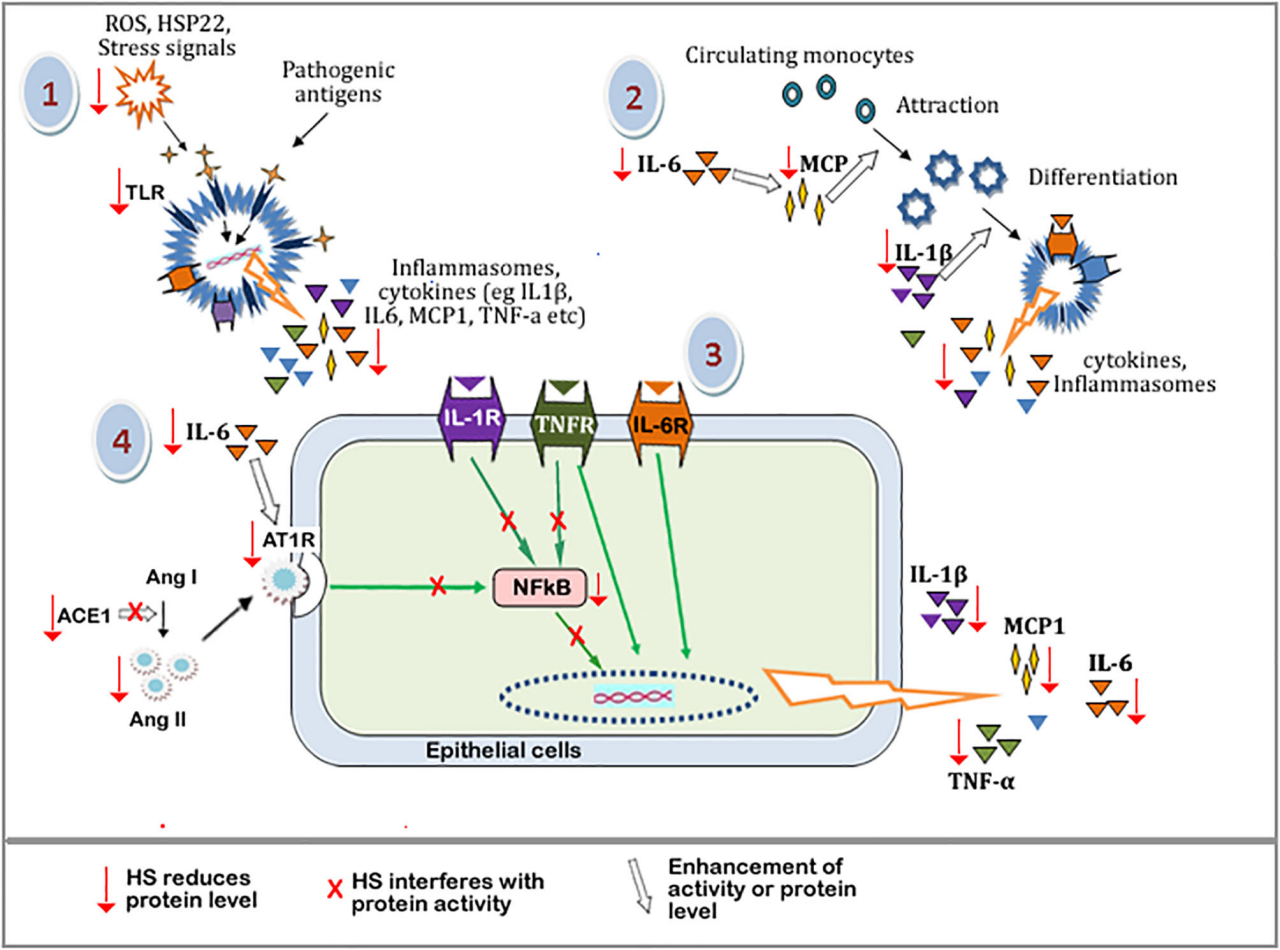
Schematic diagram showing the tentative mechanism of HS anti-inflammatory activities. Oxidative stress and other cellular damage cause the activation of TLRs, which leads to the expression of inflammatory mediators. These mediators further perpetuate immune responses causing the release of more inflammatory mediators. HS interferes with various steps along these pathways (shown by red arrow). Steps with a red arrow indicate that HS reduced the level of the component of that step. Steps with a red “X” means that HS inhibited or interfered with the protein activity along that pathway. The white arrow means that the activity or level of a component is enhanced.\ TLRs, Toll-like receptors; ROS, Reactive oxygen species; HSP22, heat shock protein 22; IL-6, Interleukin 6; IL-1b, interleukin 1b; MCP-1, Monocyte Chemoattractant Protein-1; NFkB, Nuclear factor kappa B; IL-1R, Interleukin 1 receptor; TNFR, tumor necrosis factor receptor; IL-6R, Interleukin 6 receptor; Ang I, Angiotensin I; Ang II, Angiotensin II; ACE-1, Angiotensin converting enzyme 1; AT-1R, Angiotensin II type 1 receptor.

The suggested mechanism of HS anti-inflammatory activities is outlined in **Figure 14**. The pathway leading to the buildup of inflammatory and immune responses includes: (1) build up of oxidative stress, which leads to cellular damage and the consequent release of endogenous stress signal molecules, i.e, damage-associated molecular patterns (DAMP). Stimulation by pathogens also triggers the release of pathogen-associated molecular pattern molecules (PAMP). PAMP and DAMP binding to Toll-like receptor proteins (eg TLR4) leads to the downstream expression of inflammatory mediators including MCP-1, TNF-α, IL-1β, IL-6, etc. (2) MCP-1, IL-1β, IL-6, and other cytokines trigger the accumulation and infiltration of circulating monocytes into sites of inflammation, resulting in the differentiation and maturation of the monocytes to specialized immune cells. (3) Cytokine receptors (eg TNFR, IL-6R, IL-1R) on the surface of immune or epithelial cells are activated by the binding of their respective ligands, leading to the expression of more inflammatory cytokines in an NF-κB dependent and independent pathway. (4) The activities of ACE1 lead to the buildup of Ang II. This increases the stimulation of the AT1 receptors and its associated downstream expression of inflammatory cytokines. IL-6 further enhances the expression of AT1 receptors, leading to more downstream effects.

Components of HS extracts can suppress TLR mediated expression of inflammatory mediators by reducing the levels of DAMP molecules (through their radical scavenging activities) and suppressing the expression of TLR. HS extract also suppresses the levels of MCP-1, TNF-α, IL-1β, IL-6 resulting in a reduction in the overall inflammatory/immune responses. HS extract can inhibit ACE1 activity as well as reduce the levels of ACE1 and Ang II proteins hereby suppressing downstream activation of AT1 receptor-mediated expression of inflammatory cytokines. HS can also interfere with components of NF-κB cascade thereby further suppressing the expression of inflammatory genes.

The mechanism involved in the anti-inflammatory effect of HS extract in different disease models is multifaceted and involves the interaction of different constituents of HS with different cellular targets. It appears that the mechanism that predominates depends on the causative factors of each disease model. Diseases linked to dysfunctional oxidative stress most likely could involve the antioxidant capacity enhancement potentials of HS to restore normal antioxidant levels, bringing about the cessation of the observed inflammation. In pathological conditions caused by localized inflammation, the MCP-1, COX2, and lipoxygenase suppressing properties of HS might come into play to reduce inflammation. Reduced expression of MCP-1 will suppress the infiltration of circulating monocytes into inflammatory sites, reducing the extent of the innate immune response at such sites. The ability to inhibit ACE suppresses the possibility of having an enhanced Ang II/AT1 receptor-mediated downstream oxidative dysfunction and inflammatory response. A combination of all these effects could be beneficial for suppressing immune responses in conditions of chronic systemic inflammatory disorders.

Apart from anti-inflammatory effects, other beneficial health effects have also been observed following HS supplementation. Using a combination of transcriptomics and metabolomics, Beltrán-Debón et al. (47) showed that ingestion of aqueous extract of HS triggered several components of mitochondrial functions, energy metabolism, and cardiovascular protection. Improvement of glucose tolerance, insulin sensitivity, gut integrity, and restoration of gastrointestinal microbiota was observed in experimental models of diet-induced obesity which received oral doses of HS polyphenol extracts (16). HS has been recommended for use as adjuvant therapy in the management of metabolic syndrome diseases (16), Inflammatory bowel disease (40), diabetic nephropathy (15), and wounds (60). The use of HS as an adjuvant needs to be done cautiously since HS can interact with conventional drugs such as hydrochlorothiazide, chloroquine, ciprofloxacin, and acetaminophen, as such affecting the efficacy and kinetics of the primary drug (26, 84). HS did not improve nor affect the antihypertensive potency of captopril when both were co-administered in a hypertensive rat model (26). In terms of toxicological safety, HS is reported to have a lethal dose of 50 that is greater than 5000 mg/kg weight of experimental animals (85, 86).

### Limitations

This systematic review is affected by the various limitations that are characteristics of systematic reviews. Some of such limitations include the fact that only published data that were identified using the search strategies and databases specified in our method section were accounted for in the data synthesis. The possible omission of other relevant studies either because they were not published or because they not were indexed in the databases we consulted cannot be ruled out. The data used in this review were obtained from in-vivo (animal and human) studies in a limited disease model. Furthermore, there were few studies per disease model and in some cases, conclusions were drawn using models from just two studies. Only two disease models were reported for humans (i.e hypertensive and metabolic syndrome), as such, the possible impact of the human disease model on HS efficacy could not be reliably ascertained from this study. The small number of studies (per each biomarker) made it impossible to identify the possible causes of the heterogeneity observed in some of the pooled estimates. The number of studies used to generate the pooled estimate might not be sufficient to entirely reflect the real situation. Finally, most of the data used for this systematic review and meta-analysis were obtained from studies that exposed experimental models to chronic or sub-chronic levels of HS. The instantaneous efficacy of HS administration could not be thus ascertained from this systematic review.

### Conclusion

This systematic review and meta-analysis revealed that HS extracts possess immunosuppressive capabilities. This finding suggests that HS extracts might be beneficial in the treatment/management of pathological conditions associated with a hyperactive immune system. Further studies should verify the efficacy of HS in suppressing immune responses in autoimmune or chronic inflammatory disease conditions.

## Data Availability Statement

The original contributions presented in the study are included in the article/supplementary material. Further inquiries can be directed to the corresponding author.

## Author Contributions

FU conducted the search, participated in data extraction, ran the statistical and forest plot analyses, designed the graphic abstract, and contributed to the introduction and discussion sections. JU and GB wrote the introduction and materials and methods and undertook data extraction. BE-E participated in data extraction, contributed to the discussion section, and collated the work. NC searched the databases and participated in data extraction. OO mentored the authors, reviewed the draft manuscript, and certified the final manuscript. All authors contributed to the article and approved the submitted version.

## Conflict of Interest

The authors declare that the research was conducted in the absence of any commercial or financial relationships that could be construed as a potential conflict of interest.

## Abbreviations

ACE1, Angiotensin converting enzyme -1; ACE2, Angiotensin converting enzyme -2; Ang I, Angiotensin I; Ang II , Angiotensin II; AT1 receptor, Angiotensin II receptor type 1; AT2 receptor, Angiotensin II receptor type 2; CCR2, C-C chemokine receptor type 2; COX-2, Cyclooxygenase-2; CRP, C-reactive protein; DAMP, damage-associated molecular patterns; DMBA, Dimethylbenz(A)Anthracene; ERK, Extracellular signal-regulated kinases; HepG2, Human hepatoma cell line; HFD, High fat diet; HS, Hibiscus sabdariffa calyx; ICAM-1, Intercellular Adhesion Molecule 1; IL-10, Interleukin 10; IL-1R, Interleukin-1 receptor; IL-1β, Interleukin 1β; IL-6, Interleukin 6; IL-8, Interleukin 8; iNOS, Inducible nitric oxide synthase; JNK, c-Jun N-terminal kinase; L-NAME, N(G)-nito-L-arginine-methyl ester; LPS , Lipopolysaccharide; MAP3K, Mitogen Activated Protein (MAP) kinase kinase kinase; MAPK, mitogen-activated protein kinase; MCP-1 or CCL2, Monocyte Chemoattractant Protein-1; NF-κB, Nuclear Factor Kappa B; PAMP, pathogen-associated molecular pattern molecules; PCA, protocatechuic acid; PRISMA, Preferred Reporting Items for Systematic reviews and Meta-Analysis; ROS, Reactive oxygen species; SD, standard deviation; SEM, standard error of mean; SMD, standardized mean difference; TAA, Thioacetamide; TFA, Trifluoroacetic acid; TLR, Toll like receptor; TLR4, Toll like receptor 4; TNFR, Tumor necrosis factor receptor; TNF-α, tumor necrosis factor-α; TPA, 12-O-tetradecanoylphorbol-13-acetate; TRAF2, TNF receptor associated factor 2; VCAM-1, vascular cell adhesion molecule 1; VEGF, vascular endothelial growth factor.
